# Conformation‐Modulated Lignin for Durable and High‐Output Cellulosic Triboelectric Materials Toward Self‐Powered Sensing

**DOI:** 10.1002/advs.73715

**Published:** 2026-02-09

**Authors:** Lujie Wang, Jian Du, Yilin Wang, Tianshuang Bao, Chao Li, Yehan Tao, Jinwen Hu, Chenglong Fu, Dong Lv, Weiwei Zhao, Zhanhui Yuan, Haisong Wang

**Affiliations:** ^1^ Liaoning Key Lab of Lignocellulose Chemistry and BioMaterials Liaoning Collaborative Innovation Center for Lignocellulosic Biorefinery College of Light Industry and Chemical Engineering Dalian Polytechnic University Dalian China; ^2^ College of Materials Engineering Fujian Agriculture and Forestry University Fuzhou China; ^3^ Department of Materials Science and Engineering Pohang University of Science and Technology Pohang Republic of Korea; ^4^ School of Energy and Environment City University of Hong Kong Hong Kong China; ^5^ Birmingham Centre for Energy Storage (BCES) & School of Chemical Engineering University of Birmingham Birmingham UK

**Keywords:** conformation modulation, esterified alkali lignin, self‐powered sensor, triboelectric nanogenerator

## Abstract

Cellulose‐based triboelectric sensors offer a sustainable pathway toward self‐powered electronics, yet their practical reliability is severely constrained by long‐term tribo‐induced fiber fibrillation and the resulting interfacial degradation. Herein, we report a conformation‐engineered alkali lignin (EAL) that enables simultaneous enhancement of mechanical durability and electrical output in cellulose‐based triboelectric materials. Succinic‐anhydride grafting introduces flexible aliphatic side chains into the rigid lignin backbone, granting dynamic conformational adaptability that promotes chain sliding and efficient frictional energy dissipation. This molecular strategy significantly suppresses interfacial wear, reducing the wear rate of the EAL/Cellulose composite by 52.44% after 5000 cycles. Conformation modulation also improves dielectric polarization, leading to markedly increased triboelectric performance, with the optimized device delivering high Voc, Isc, and Qsc values of 100 V, 5.26 µA, and 56.87 nC. The EAL/Cellulose‐based sensor exhibits high signal fidelity in robotic finger sliding detection and handwriting recognition, and maintains stable performance after 500 times repeated writing. In addition, the EAL/Cellulose composite demonstrates strong recyclability in a natural environment and rapid enzymatic degradability within 24 h. This work presented a conformation‐guided molecular design strategy for developing durable, high‐output, and eco‐friendly cellulose‐based triboelectric devices for next‐generation self‐powered sensing.

## Graphical abstract

Conformation‐engineered alkali lignin with flexible aliphatic side chains is integrated into cellulose to suppress tribo‐induced fiber fibrillation and interfacial wear. Dynamic chain adaptability enhances frictional energy dissipation and dielectric polarization, enabling durable, high‐output triboelectric sensing with excellent recyclability and rapid biodegradability for sustainable self‐powered electronics.

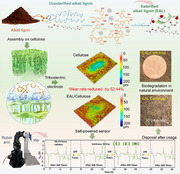

## 1 Introduction

The rapid growth of self‐powered sensing systems in wearable electronics, intelligent robotics, and distributed Internet‐of‐Things (IoT) networks has intensified the demand for light‐weight, sustainable, and mechanically robust triboelectric materials [1]. Triboelectric nanogenerators (TENGs), which harvest mechanical energy through contact electrification and electrostatic induction, have emerged as a promising platform for powering next‐generation sensing devices [2, 3]. Among various green triboelectric materials, cellulose has drawn widespread attention due to its renewability, flexibility, and compatibility with scalable paper‐based manufacturing. However, despite these advantages, cellulose‐based TENGs still face two long‐standing challenges that critically restrict their durability and signal stability [4]. First, mechanical wear‐induced fibrillation occurred easily during repeated contact‐separation, leading to structural disruption of cellulose fibers and progressive exposure of hydroxyl‐rich microfibrils [5]. Such interfacial damage not only weakens the mechanical integrity of the tribolayer but also accelerates charge dissipation, especially under humid conditions, resulting in substantial output decay. Second, enhancing the triboelectric output of cellulose still remains complex because its relatively weak dielectric polarization limits charge transfer [6]. Existing improvement strategies, such as ionic lubrication, hierarchical surface structuring, or nanofillers reinforcement, offer partial benefits but generally require complex fabrication or fail to reconcile the conflicting requirements of mechanical durability, humidity stability, and high triboelectric performance [7].

Lignin, the second most abundant natural polymer, offers an attractive opportunity for simultaneously improving the wear resistance and dielectric properties of cellulose‐based triboelectric materials [8]. Its aromatic backbone, phenolic hydroxyl groups, and polar functionalities give lignin intrinsic dielectric advantages and strong interfacial interactions with cellulose. Prior studies have explored lignin as a lubricant additive or nanoadditives to mitigate friction at the macroscopic scale, not directly used as the triboelectric layer [9]. However, when lignin is directly coated onto cellulose, its unmodified rigid aromatic backbone severely limits molecular mobility, resulting in stress concentration and interfacial abrasion during high‐frequency sliding, which represents a critical yet often overlooked challenge for triboelectric interfaces [10]. In addition, the relatively low triboelectric polarity of lignin is not beneficial for the improvement of output signals of TENG [11]. To overcome these drawbacks, a promising strategy is molecular conformation engineering. Introducing flexible aliphatic side chains into lignin via esterification can tune the rigidity of its backbone, enhance segmental mobility, and enable dynamic chain rearrangement under shear [12]. Such conformational adaptability not only facilitates efficient mechanical energy dissipation and suppression of friction‐induced fiber damage but also increases dielectric polarization and dipole moment, enhancing charge generation during contact electrification. Despite its conceptual potential, the conformational regulation of lignin for integrated mechanical durability and triboelectric enhancement has not been systematically explored [13].

In this work, we introduced a conformation‐engineered esterified alkali lignin (EAL) as a multifunctional molecular modifier for cellulose‐based triboelectric systems. By grafting succinic‐anhydride‐derived flexible side chains, we tailor lignin's conformational flexibility and interfacial compatibility to construct a robust cellulose skeleton‐lignin network with superior wear resistance and enhanced dielectric characteristics. We systematically elucidate how conformation modulation governs triboelectric energy dissipation, dielectric behavior, output stability, and sensing performance. The optimized EAL/Cellulose composite achieves reduced wear under repeated friction, increased dielectric constant and surface potential, stable output under high humidity, and high‐resolution self‐powered sensing of robotic motion and handwriting. This conformation‐guided strategy establishes a molecular design principle that addresses the dual challenges of durability and high output while maintaining sustainability, paving the way for next‐generation cellulose‐based materials for self‐powered flexible electronics.

## 2 Results and Discussion

### 2.1 Esterification of Alkali Lignin

**FIGURE 1:**
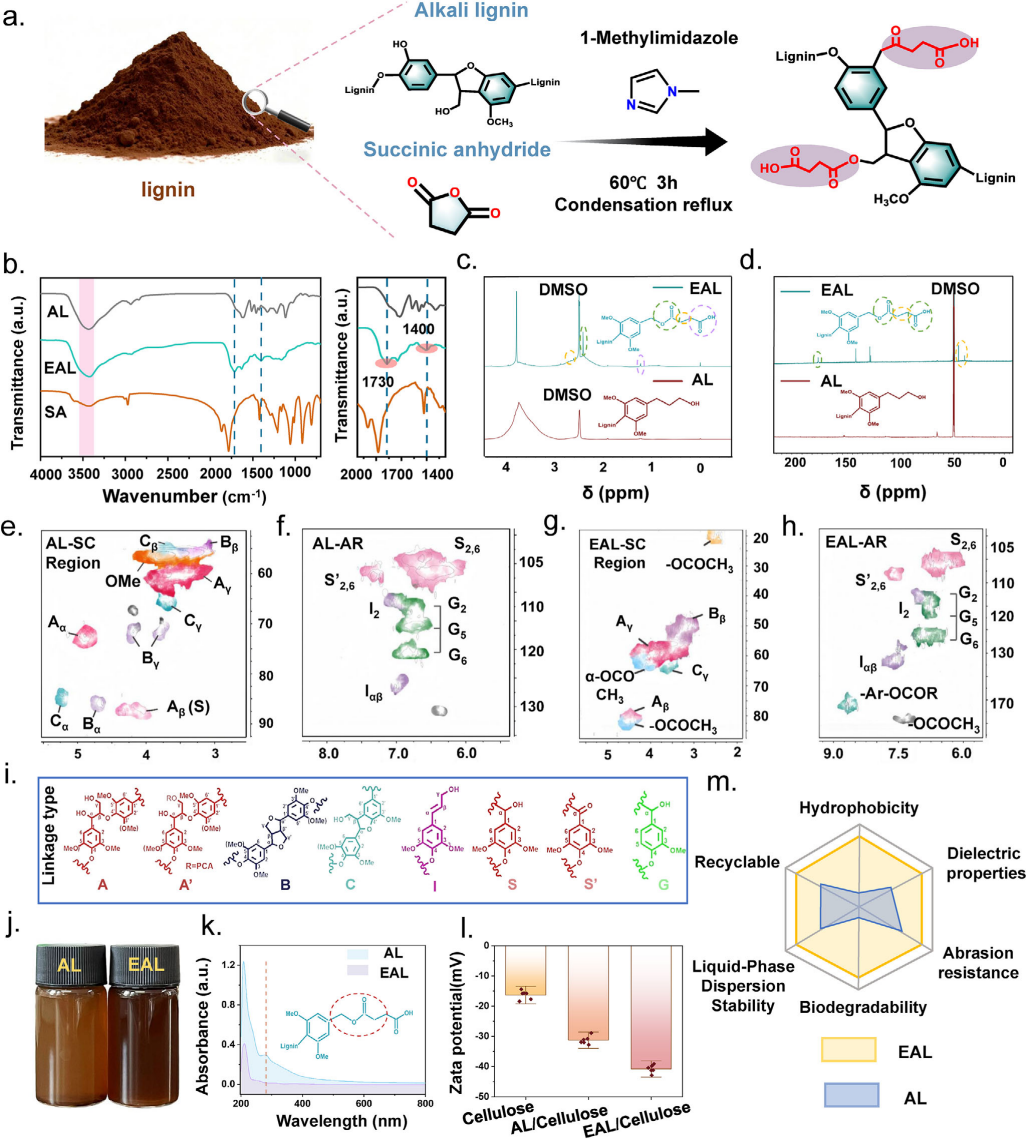
(a) Preparation process of esterified alkali lignin (EAL). (b) FTIR spectra of AL, EAL, and SA. (c) ^1^H NMR spectra of AL and EAL. (d) ^13^C NMR spectra of AL and EAL. (e, f) 2D NMR spectra of AL in the side‐chain (SC) and aromatic (AR) regions. (g, h) 2D NMR spectra of EAL in the SC and AR regions. (i) Schematic illustration of linkage types before and after esterification. (j) Homogeneous dispersions of AL and EAL. (k) UV–vis spectra of AL and EAL. (l) Zeta potentials of cellulose, AL, and EAL. (m) Radar chart comparing the key properties of AL and EAL.

As illustrated in Figure 1a, alkali lignin (AL) was selected as the precursor, and its native phenolic hydroxyl and methoxy groups served as active reaction sites for succinic anhydride (SA)‐based esterification. During the modification process, 1‐methylimidazole (1‐MI) was introduced as an auxiliary component. Owing to its mild basicity and good polarity, 1‐MIM facilitates the activation of phenolic hydroxyl groups and improves the dispersion of AL in the reaction medium. This enables more uniform contact between SA and lignin molecules and allows efficient and homogeneous esterification under mild conditions (60°C, 3 h), while preserving the integrity of the lignin aromatic backbone. Successful incorporation of ester groups was first confirmed by FTIR spectroscopy (Figure 1b). After esterification, EAL exhibits a new C═O stretching band at 1730 cm^−1^ and a characteristic methylene bending vibration of the succinyl group at 1400 cm^−1^. ^1^H NMR spectra (Figure 1c) reveal a new peak at 2.7 ppm attributed to the methylene protons of the succinate moiety, along with additional peaks at 2.4 and 1.9 ppm corresponding to aromatic and aliphatic acetyl protons. In the ^13^C NMR spectrum (Figure 1d), the intensified signal near 175 ppm further verifies the formation of ester and carboxyl carbonyls. 2D NMR analysis (Figure 1e–i) clearly identifies the succinyl cross‐peak (δ_C_/δ_H_ ≈ 29/2.65) and a ^3^JCH correlation between the ester carbonyl carbon (δ_C_ ≈ 172 ppm) and aromatic protons, confirming that the succinyl group is covalently grafted onto the lignin aromatic ring through the phenoxy site. UV spectra (Figure 1j–k) show a marked decrease in the 280 nm absorption band, indicating partial consumption of phenolic hydroxyl groups and further supporting successful esterification. XRD patterns (Figure S1) show weakened and broadened characteristic peaks after esterification, suggesting decreased molecular ordering and altered packing due to the introduction of flexible side chains. Zeta‐potential analysis (Figure 1l) reveals that EAL carries a significantly higher negative charge (ζ ≈ −42 mV) compared with cellulose (−15 mV) and unmodified AL (−30 mV), demonstrating markedly improved dispersion stability in aqueous media. Finally, the radar chart in Figure 1m highlights the comprehensive performance enhancement of EAL. Compared with AL, EAL shows superior hydrophobicity, dielectric properties, dispersion stability, biodegradability, recyclability, and wear resistance, establishing esterification as an effective strategy for upgrading lignin into a multifunctional material suitable for triboelectric applications [14].

### 2.2 Characterizations of Wear‐Resistant Cellulosic Paper

**FIGURE 2:**
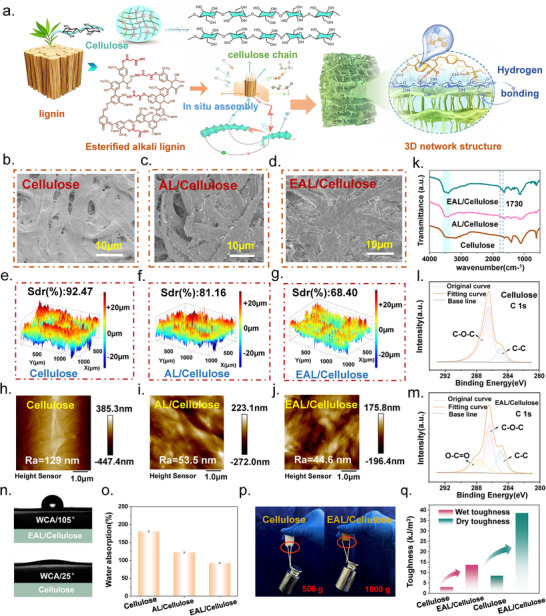
(a) Schematic illustration of the self‐assembly strategy for constructing EAL/Cellulose composites. (b–d) SEM images of Cellulose, AL/Cellulose, and EAL/Cellulose, respectively. (e–g) 3D surface morphologies of Cellulose, AL/Cellulose, and EAL/Cellulose films prior to testing. (h–j) AFM images of Cellulose, AL/Cellulose, and EAL/Cellulose. (k) FTIR spectra of Cellulose, AL/Cellulose, and EAL/Cellulose. (l,m) High‐resolution C 1s XPS spectra of Cellulose and EAL/Cellulose. (n) Water contact angles of AL/Cellulose and EAL/Cellulose. (o) Water absorption/swelling ratios of Cellulose, AL/Cellulose, and EAL/Cellulose. (p) Maximum load‐bearing capacity of Cellulose and EAL/Cellulose. (q) Dry and wet toughness of Cellulose and EAL/Cellulose.

Through the layer‐by‐layer self‐assembly strategy (Figure 2a and Figure S2), the oxygen‐containing functional groups in EAL, such as ester groups (‐C(O)OR) and phenolic hydroxyls (‐ArOH), form multiple hydrogen‐bonding interactions with the abundant ‐OH groups on cellulose fibers [15]. These intermolecular interactions enable the in situ anchoring of EAL onto the cellulose framework, yielding a stable “cellulose skeleton‐EAL network” structure [16]. Such hydrogen‐bond‐dominated interfacial assembly effectively overcomes the inherent incompatibility between lignin and cellulose and provides a robust structural basis for the enhanced mechanical integrity and triboelectric stability of the composite. The microstructural evolution observed by SEM further confirms the interfacial assembly behavior (Figure 2b–d and Figure S3). In the AL/Cellulose composite, noticeable lignin particle agglomerates are present on the surface, accompanied by interfacial gaps between lignin clusters and cellulose fibers, indicating relatively poor interfacial interaction of unmodified lignin. In contrast, the EAL/Cellulose surface exhibits a continuous and uniform coating without visible particle aggregation, demonstrating that EAL spreads more evenly and tightly adheres to the cellulose fibers. Cross‐sectional SEM images (Figure S4) show that EAL induces closer fiber packing, causing slight structural shrinkage and a reduced overall thickness, which suggests enhanced inter‐fiber bonding and network densification. From pristine cellulose to the EAL/cellulose composite, the 3D surface morphology becomes progressively smoother, as shown in Figure 2e–g. This trend is consistent with the 2D surface images, where the EAL/cellulose sample exhibits a more uniform topography (Figure S5). The surface roughness (Sdr) also decreases markedly from 92.47% to 68.40%. AFM measurements further reveal a clear gradient reduction in microscopic roughness (Figure 2h–j), with the Ra value decreasing from 129 nm for pristine cellulose to 44.6 nm for EAL/cellulose. These results indicate that the incorporation of EAL reconstructs the microstructure of cellulose and transforms the surface from a porous and loose state into a dense and uniform one. This morphology regulation enhances surface homogeneity by reducing interfacial voids. The elimination of stress concentration and the suppression of fiber damage significantly improve the durability of the material. To ensure consistent material performance, we systematically evaluated the uniformity of the composite‐layer thickness and the reproducibility of its fabrication. As shown in Figure S6, individual samples exhibit highly uniform thickness with no noticeable regional variation, and consecutive batches show nearly identical average thicknesses. These results verify the excellent thickness uniformity of the composite layer and the stability of the fabrication process, ensuring highly reproducible material properties. The FTIR spectra provide further molecular interaction between EAL and cellulose after assembly (Figure 2k). The characteristic ester carbonyl peak at 1730 cm^−1^ in the EAL/Cellulose composite confirms the presence of EAL on the cellulose surface. Meanwhile, no new peaks appear in the composite spectrum, indicating that the interaction between EAL and cellulose is governed primarily by physical forces, such as hydrogen bonding and van der Waals interactions, rather than the formation of new covalent bonds [17]. These results are fully consistent with the homogeneous coating observed in SEM and collectively confirm the excellent interfacial compatibility and self‐assembly behavior of esterified lignin on cellulose fibers [18].

The XPS analysis provides deeper insight into how lignin and esterified lignin interact with the cellulose surface at the molecular level (Figure 2l, m and Figures S7 and S8). Pristine cellulose displays a C 1s spectrum dominated by C─O─C and C─O species, reflecting its polysaccharide backbone. After the introduction of AL, the increased contribution of the C─C component indicates that aromatic and aliphatic carbons from lignin become exposed at the interface, suggesting that lignin coats or partially replaces the surface cellulose chains. This shift implies that *π–π* interactions and hydrogen bonding between lignin and cellulose facilitate their interfacial assembly, leading to a heterogeneous carbon environment that differs from pure cellulose. For the EAL/Cellulose composite, the emergence of a distinct C═O signal provides direct evidence of ester functionalities originating from EAL. The presence of ester carbonyls on the cellulose surface demonstrates not only the successful grafting of succinyl groups onto lignin but also their effective exposure at the composite interface. This carbonyl enrichment suggests that EAL possesses enhanced interfacial affinity toward cellulose, likely due to its modified polarity and the ability of ester groups to participate in dipole–dipole interactions and reinforced hydrogen‐bond networks. As a result, the cellulose surface transitions from a hydroxyl‐rich environment to a more complex chemical interface containing aromatic, aliphatic, and carbonyl‐bearing groups. The evolution of C─C, C─O─C/C─O, and C═O species across the three samples confirms that lignin, especially esterified lignin, reorganizes the cellulose surface chemistry. This interfacial reconstruction provides the molecular basis for enhanced dielectric properties, stronger interfacial adhesion, and improved frictional stability observed in the EAL/Cellulose composites [19].

To elucidate the interfacial regulation enabled by EAL, we first examined the surface wetting behavior (Figure 2n and Figure S9). Pristine cellulose exhibits strong hydrophilicity with a water contact angle of 25^°^, whereas the EAL/Cellulose composite shows a water contact angle of 105^°^, indicating that cellulose fibers are uniformly coated by a low‐polarity ester‐rich layer. This hydrophobic barrier effectively suppresses water infiltration into the fiber network, resulting in a markedly reduced swelling ratio compared with unmodified samples (Figure 2o). The excellent moisture resistance stems from a key chemical modification strategy that converts lignin's hydrophilic hydroxyl groups into hydrophobic ester bonds, substantially reducing intrinsic hydrophilicity. During layer‐by‐layer assembly, the modified lignin and cellulose form hydrogen‐bonded coatings that further shield hydroxyl groups, thereby markedly lowering the hydrophilicity of the EAL/cellulose triboelectric electrode. Such interfacial modulation also translates into pronounced mechanical enhancement. The EAL/Cellulose composite displays significantly higher dry and wet toughness, and is capable of sustaining a 1000 g suspended load without fracture (Figure 2p). The substantial increase in both dry toughness (from 8.48 to 38.57 kJ m^−3^) and wet toughness (from 2.93 to 13.66 kJ m^−3^) further demonstrates that the EAL‐reinforced hydrogen‐bonding network effectively dissipates mechanical energy and resists water‐induced softening (Figure 2q and Figure S10). This dual strengthening mechanism enables the composite to maintain a high load‐bearing capacity even under humid conditions. We further assessed the interfacial stability of the material under extreme conditions using an ultrasonic durability test. As shown in Figure S11, the EAL/Cellulose composite exhibited no delamination or structural damage after prolonged ultrasonication, nor were any noticeable changes observed in the solution. These results demonstrate the strong molecular‐level adhesion between EAL and cellulose, enabling the composite to withstand severe mechanical perturbation.

### 2.3 Triboelectric Output Performances

**FIGURE 3:**
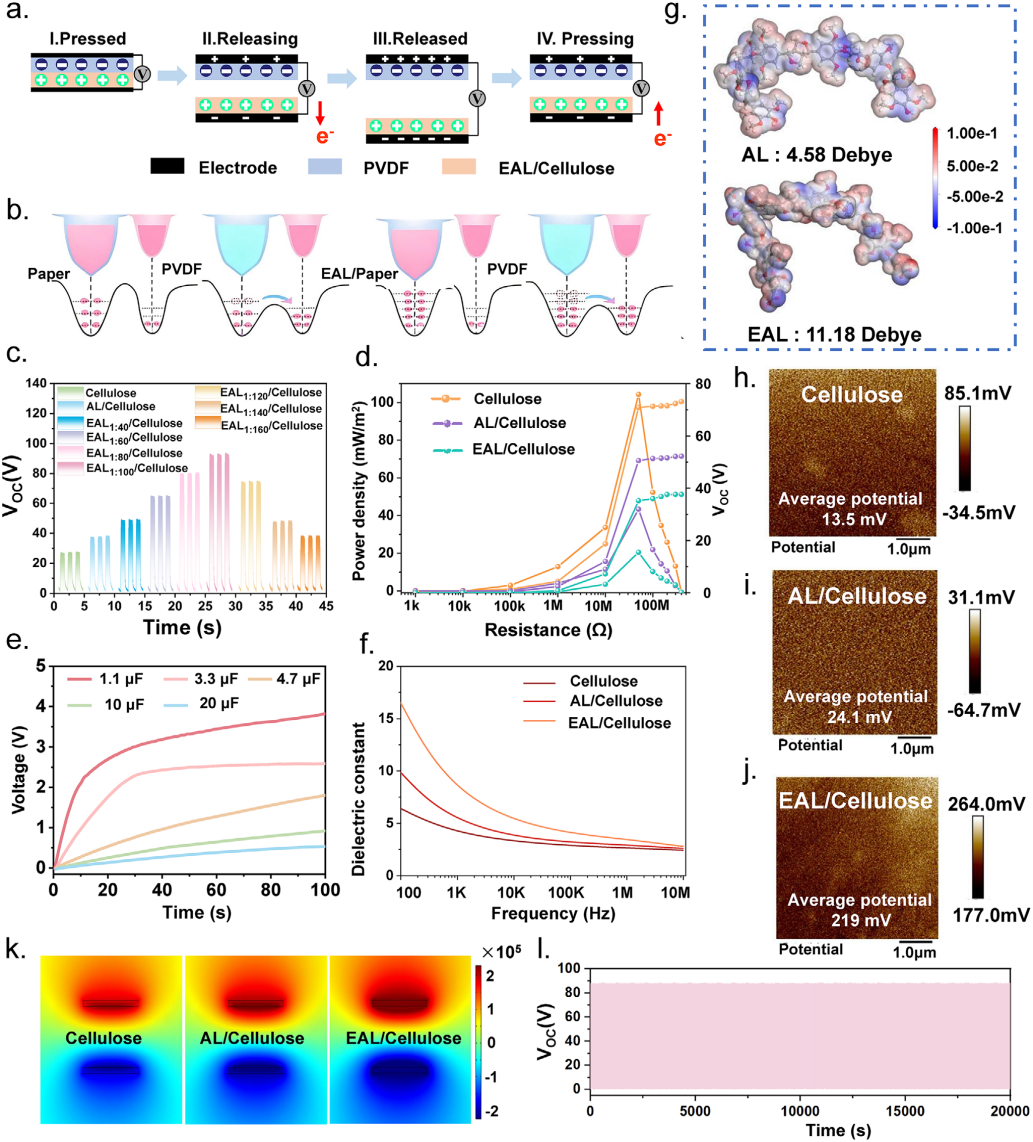
(a) Working mechanism of the cellulosic TENG. (b) Schematic illustration of the charge‐transfer process during contact‐separation. (c) V_OC_ of cellulose‐based triboelectric layers with different AL and EAL ratios. (d) Output power densities of Cellulose, AL/Cellulose, and EAL/Cellulose TENGs. (e) Charging performance of the optimized EAL/Cellulose TENG under different capacitor values. (f) Dielectric properties of Cellulose, AL/Cellulose, and EAL/Cellulose. (g) Simulated dipole moments of AL and EAL. (h‐j) Surface potential mapping of Cellulose, AL/Cellulose, and EAL/Cellulose, respectively. (k) COMSOL‐simulated electric potential distributions of Cellulose, AL/Cellulose, and EAL/Cellulose (top) contacting PVDF (bottom). (l) Long‐term output stability of the EAL/Cellulose TENG after 20000 operation cycles.

In this study, an Ag/PVDF‖EAL/Cellulose/Ag triboelectric nanogenerator (TENG) was constructed (Figure 3a, b and Figure S12), operating in the vertical contact‐separation mode to convert mechanical motion into electricity [20]. The working mechanism originates from the coupling of contact electrification and electrostatic induction. When the EAL/Cellulose tribo‐layer comes into contact with the PVDF layer, differences in their intrinsic triboelectric polarities lead to charge transfer at the interface, resulting in opposite and balanced surface charges on the two materials. Upon separation, the interfacial charges remain immobilized, establishing an electrostatic field that induces a potential difference between the top and bottom electrodes. This potential drives electrons through the external circuit to balance the electrostatic field. When the layers approach and contact again, the reduced separation distance diminishes the induced potential, causing electrons to flow back in the opposite direction. Through repeated contact‐separation cycles, the device continuously outputs alternating voltage and current, enabling efficient harvesting of biomechanical energy [21].

To clarify the regulatory effects of pure cellulose, AL/Cellulose, and EAL/Cellulose on the triboelectric output, we systematically measured the open‐circuit voltage (V_OC_), short‐circuit current (I_SC_), and transferred charge (Q_SC_) of TENGs constructed with different triboelectric layers (Figure 3c and Figure S13). The pristine cellulose‐based TENG exhibits a V_OC_ of 25 V, an Isc of 2.12 µA, and a Qsc of 16.23 nC. After introducing unmodified lignin (AL), all three output parameters increase to 40 V, 2.67 µA, and 23.41 nC, respectively, confirming that the additional polar groups in AL facilitate surface charge generation. More importantly, by tuning the succinic‐anhydride grafting level, the electrical output of the EAL/Cellulose TENG can be further optimized. Among all ratios tested, the EAL_1:100_/Cellulose device delivers the highest triboelectric performance, achieving a Voc of 100 V, an Isc of 5.26 µA, and a Qsc of 56.87 nC. In addition, the output characteristics and instantaneous power of the devices were evaluated by connecting external load resistances ranging from 1 kΩ to 400 MΩ (Figure 3d). As the load resistance increased, the V_OC_ of all three TENGs gradually rose and reached a relatively equilibrium state when the resistance exceeded 30 MΩ. Consequently, the maximum power output appears at an intermediate resistance. As shown in Figure S14, the AL/Cellulose‐based TENG exhibits its peak power density of 31.96 mW m^−2^ at approximately 30 MΩ. The EAL/Cellulose‐based TENG delivers an even higher peak power density of 75.89 mW m^−2^, representing an enhancement of about 79.6% compared with the pure cellulose TENG (15.48 mW m^−2^). These results demonstrate that the incorporation of EAL significantly improves the electrical output capability of the composite triboelectric layer [22]. After rectification, the TENG can serve as a sustainable power source and rapidly charge a 1.1 µF capacitor to 4.02 V within 100 s (Figure 3e). In addition, the optimized EAL/Cellulose‐based TENG exhibits superior output performance compared with most previously reported cellulose‐based TENGs (Table S1) as well as triboelectric devices constructed from wear‐resistant triboelectric layers (Table S2).

To elucidate the underlying mechanism responsible for the enhanced triboelectric output, comprehensive dielectric analysis, dipole‐moment simulations, and surface potential measurements were carried out [23, 24, 25]. In a TENG, the ability of a triboelectric layer to donate or capture electrons is closely associated with its dielectric polarization. As shown in the frequency‐dependent dielectric spectra (Figure 3f and Figure S15), at 100 Hz, the dielectric constant of pristine cellulose is 6.58. This value increases to 10.21 for AL/Cellulose and further rises to 17.86 for EAL/Cellulose, corresponding to a 2.72 fold enhancement relative to cellulose. The significant increase in dielectric permittivity arises from the esterification‐induced molecular conformation flexibility, which promotes segmental mobility and facilitates dipolar and interfacial polarization, thereby strengthening charge storage and boosting triboelectric output. Theoretical dipole‐moment simulations (Figure 3g) provide additional molecular‐level evidence. Owing to the introduction of polar ester groups (‐COO^−^), EAL exhibits a markedly higher dipole moment (11.18 Debye) compared with unmodified AL (4.58 Debye). The enhanced dipole moment improves the orientation polarization of EAL molecules under the triboelectric field, facilitating electron transfer toward PVDF and resulting in a higher surface charge density. This trend is fully consistent with the experimentally observed increase in V_OC_ and power density. Surface Kelvin probe force microscopy (KPFM) further validates these findings (Figure 3h–j). The average surface potential of AL/Cellulose reaches 24.1 mV, approximately 1.78 times that of pristine cellulose (13.5 mV). Remarkably, EAL/Cellulose exhibits a much higher surface potential of 219 mV (16.2 times that of cellulose), indicating a substantially stronger electron‐donating tendency during contact electrification. Such pronounced surface potential elevation directly supports the enhanced triboelectric charge generation [26]. A simplified finite‐element analysis using COMSOL (Figure 3k) visually confirms these trends. As the dielectric property improves from Cellulose to AL/Cellulose and ultimately EAL/Cellulose, the electrostatic potential in the positive triboelectric layer gradually shifts to deeper red regions, indicative of a strengthened local electric field. This simulation provides theoretical support for the dielectric‐constant‐mediated enhancement of triboelectric performance. More importantly, the EAL/Cellulose‐based TENG demonstrates exceptional long‐term durability. As shown in Figure 3l, the output voltage remains highly stable even after 20 000 consecutive operation cycles, reflecting the excellent wear resistance and interfacial robustness of the EAL‐modified triboelectric layer. These results highlight the strong potential of EAL/Cellulose for reliable, long‐term self‐powered sensing applications [27].

### 2.4 Wear‐Resistance Performance and Mechanistic Insights

**FIGURE 4:**
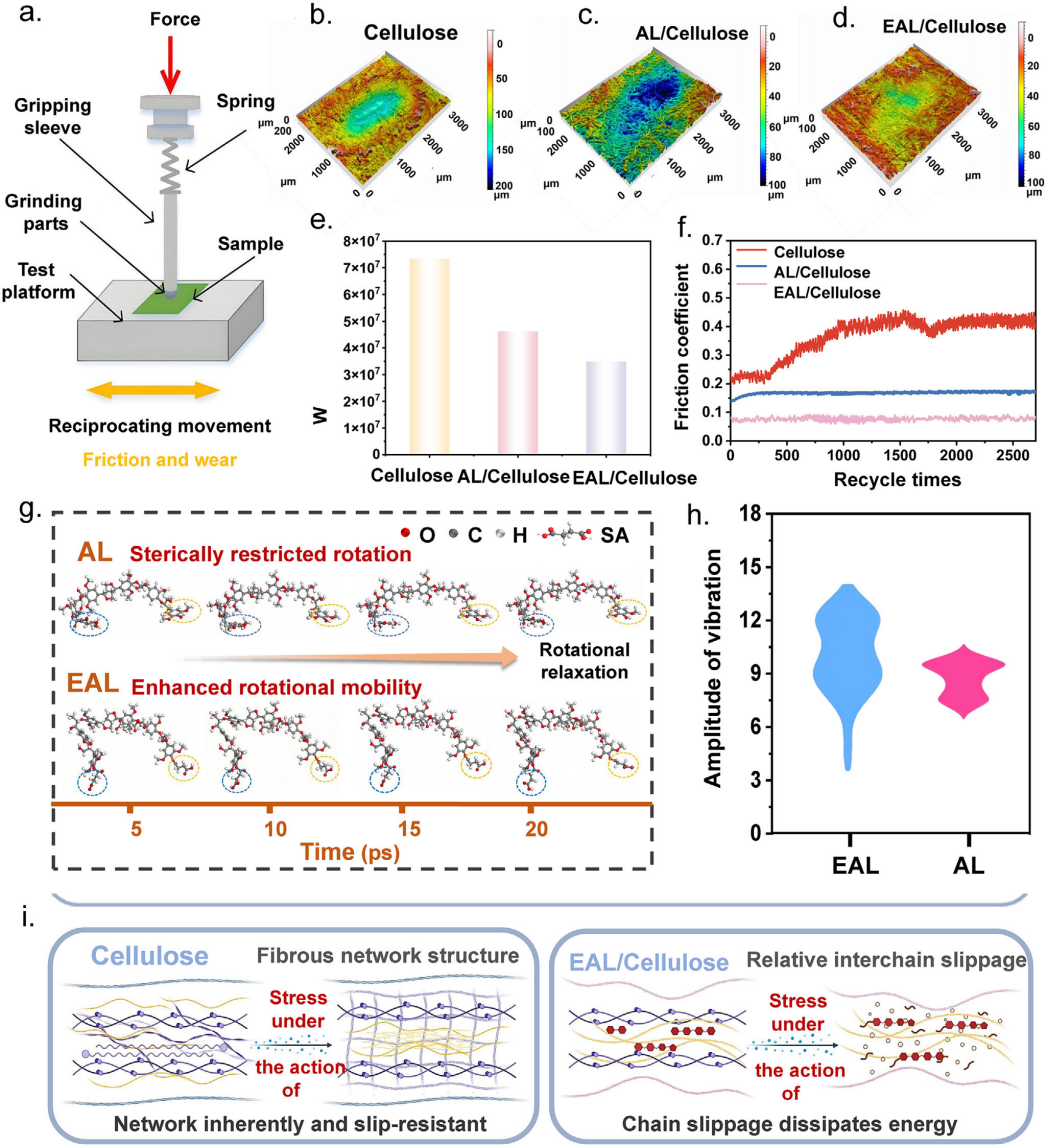
(a) Schematic illustration of the reciprocating friction test apparatus operated under a 3 N normal load and 3 Hz frequency. (b–d) 3D surface topographies of Cellulose, AL/Cellulose, and EAL/Cellulose films after 5000 friction cycles. (e) Comparison of wear volume for Cellulose, AL/Cellulose, and EAL/Cellulose films after 5000 cycles. (f) Friction coefficient chart of Cellulose, AL/Cellulose, EAL/Cellulose. (g) Schematic illustration of the conformational transition between AL and EAL molecular structures. (h) Vibration amplitude profiles of AL and EAL demonstrating enhanced molecular flexibility after esterification. (i) Proposed schematic of the stress‐response and anti‐wear mechanism of Cellulose and EAL/Cellulose composites.

In general, pure cellulose‐based triboelectric materials tend to experience fibrillation during friction, leading to structural damage caused by hydrogen bond rupture and molecular chain detachment [28]. In this study, the esterified lignin (EAL) with a flexible molecular structure forms a stable hydrogen‐bonded crosslinking network with cellulose, which can withstand mechanical stress and in situ generate a continuous and compact protective layer during friction. This structural evolution endows the EAL/Cellulose‐based TENG with superior wear resistance and long‐term durability. To systematically assess the wear resistance of the composite films, a reciprocating friction test was performed using the setup shown in Figure 4a under ambient conditions with a 3 N normal load and 3 Hz frequency for 5000 cycles. After friction (Figure 4b–d and Figures S16–S18), the EAL/Cellulose film retained the most continuous and shallow wear track, with a wear volume of 0.0348 mm^3^, approximately 52.5% lower than that of pure cellulose (0.0732 mm^3^), and a friction coefficient below 0.1 (Figure 4e, f), highlighting its superior antiwear and friction‐reducing performance. Mechanistically, the esterified lignin (EAL) structure attenuates intermolecular hydrogen bonding and dipole‐dipole interactions, while the flexible ester linkages and hydrophobic chains enhance molecular mobility and conformational adaptability, allowing efficient dissipation of shear energy through intramolecular relaxation and dynamic rearrangement during sliding (Figure 4g). The esterification modification endows EAL molecular chains with greater mobility, as evidenced by the significantly larger vibration amplitude compared with AL (Figure 4h). Under external mechanical stress, the flexible ester groups introduced by the modification effectively weaken intermolecular interactions, allowing the fibrous network to undergo relaxation and structural rearrangement. Consequently, EAL molecular chains exhibit easier slippage and directional reorganization, facilitating dynamic energy dissipation and enhancing the overall adaptability of the network under friction. As illustrated in Figure 4i, such conformational reconstruction not only facilitates the formation of a dynamic and self‐adaptive in situ frictional protection layer but also enables efficient energy dissipation through molecular chain slippage and entanglement. This process effectively alleviates localized stress concentration and suppresses the fracture and delamination of the cellulose matrix. Consequently, the modified fibrous network imparts superior frictional adaptability and antiwear capability to the composite, leading to a remarkable enhancement in overall wear resistance [29]. This work improves cellulose wear resistance by grafting tailored organic molecules onto lignin, leveraging its abundant reactive sites to introduce interaction‐enhancing or flexibility‐imparting groups. We believe that this strategy is broadly generalizable to organic systems compatible with esterification, amidation, etherification, and related grafting reactions.

**FIGURE 5:**
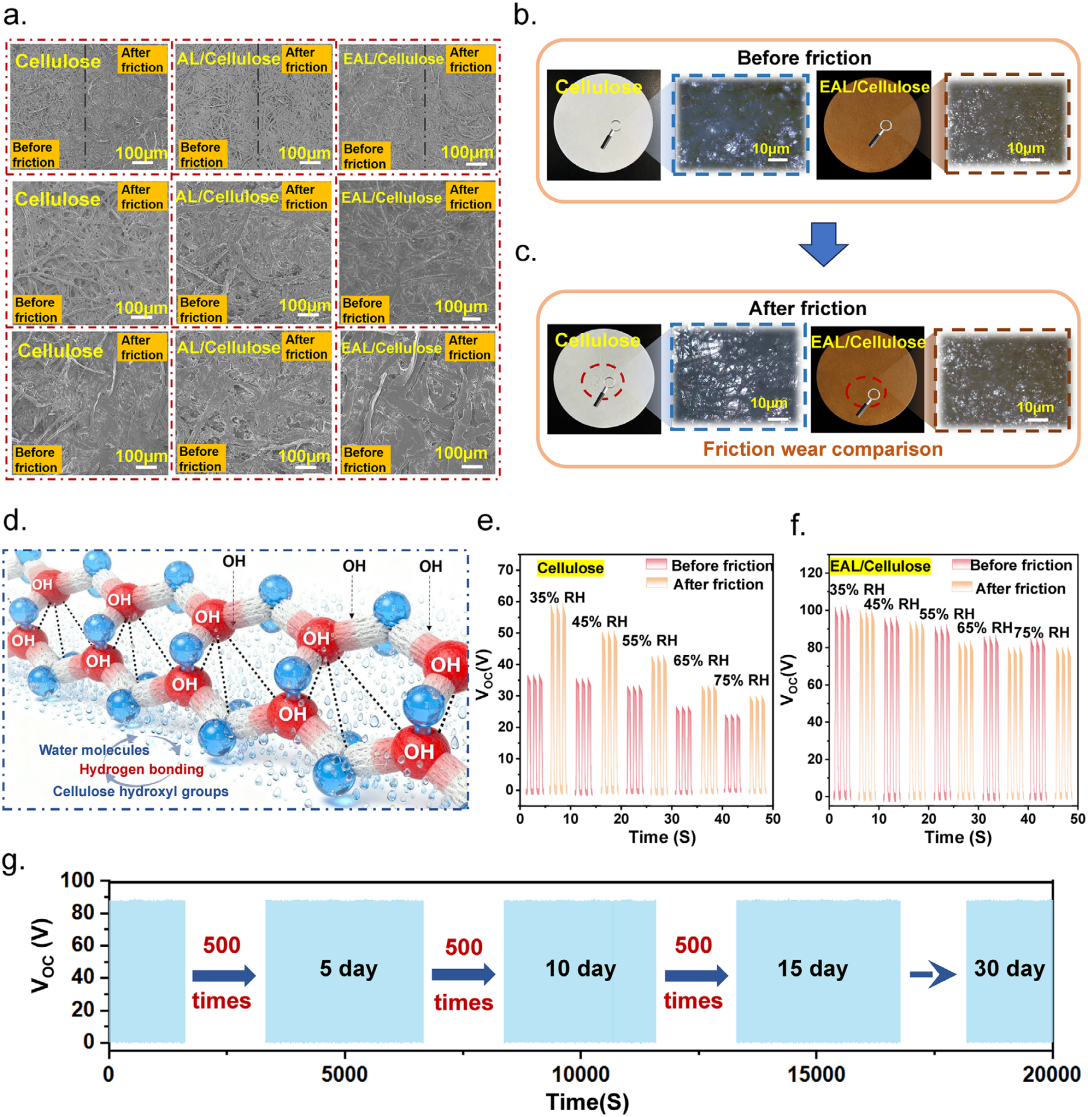
(a) High‐magnification SEM images of Cellulose, AL/Cellulose, and EAL/Cellulose before and after friction. (b) Low‐magnification optical microscope images of Cellulose and EAL/Cellulose before friction. (c) Low‐magnification optical microscope images of Cellulose and EAL/Cellulose after friction. (d) Humidity sensitivity map of Cellulose enriched in ‐OH groups. (e) Humidity‐dependent output signals of Cellulose before and after friction. (f) Humidity‐dependent output signals of EAL/Cellulose before and after friction. (g) Thirty‐day storage stability of EAL/Cellulose.

After frictional testing, the SEM images (Figure 5a) show that the structure of the pure cellulose filter paper becomes markedly loosened, with numerous fine fibrillar fragments generated along the primary fiber skeleton [30]. This indicates that the material is prone to breakage and peeling under mechanical stress, exhibiting poor wear resistance. The AL/Cellulose composite demonstrates a partially improved structural integrity compared with pristine cellulose after friction; however, evident fracture traces and small debris are still present along the main fiber backbone, suggesting that a complete protective surface layer has not formed. In contrast, the EAL/Cellulose composite exhibits no noticeable fiber breakage or debris generation after friction, and its surface remains relatively intact, confirming that esterification modification effectively reinforces the stability of the fiber network. Further microscopic observations before and after friction (Figure 5b, c) reveal that pure cellulose fibers develop prominent porous features after friction, whereas the EAL molecular chains form a dense and uniform in situ frictional protection layer on the cellulose surface, substantially enhancing the compactness and wear resistance of the material [31].

The environmental stability of triboelectric materials is crucial for practical applications, especially under complex real‐world conditions, such as cloudy weather, rainfall, or marine environments with high humidity [32]. Under such conditions, interfacial damage is more susceptible to environmental disturbances, leading to signal instability and compromising the reliability of data acquisition. Due to the high density of exposed hydroxyl groups (‐OH) on cellulose, numerous water molecules tend to adsorb onto its surface under humid conditions. During subsequent evaporation, these water molecules disrupt the stability of interfacial charges, accelerating the dissipation of triboelectric charges (Figure 5d) and resulting in a significant reduction in output signals. Therefore, we evaluated the changes in device output under different humidity levels before and after friction to further verify the role of EAL in enhancing anti‐friction performance. As shown in Figure 5e, f, when the relative humidity increases from 35% RH to 75% RH, the V_OC_ of the pure cellulose TENG decreases from 37.21 to 26.45 V, corresponding to a reduction of 28.92%, attributable to charge dissipation caused by water evaporation. After 100 friction cycles, the TENG output increases to 59.78 V under 35% RH. This enhancement primarily arises from friction‐induced fibrillation of cellulose, which exposes additional hydroxyl groups that were previously embedded within the structure. The increased availability of these active triboelectric sites facilitates more efficient charge transfer, ultimately leading to the elevated TENG output after friction. However, when the humidity rises from 35% RH to 75% RH, the V_OC_ decreases from 59.78 to 32.28 V, with a drop of 46.07%, substantially greater than that of the pristine cellulose TENG. In contrast, for the EAL/Cellulose TENG, the V_OC_ before and after friction decreases from 100 and 99.78 V to 95.55 and 94.98 V, corresponding to reductions of 4.45% and 4.81%, respectively. These results indicate that the assembly of the EAL layer on the cellulose surface effectively suppresses friction‐induced fibrillation and the generation of fine fibers, thereby improving the wear‐resistance of cellulose‐based TENGs. This enhancement ensures the stability and reliability of signal acquisition for cellulose TENGs in complex high‐humidity environments. To evaluate the influence of long‐term storage on output performance, we further conducted a storage stability test under a dry ambient condition (25°C, 35% RH). The output voltage of the EAL/Cellulose TENG was characterized after storage for 5, 10, 15, and 30 days. As shown in Figure 5g, the material maintains stable electrical output throughout the entire storage period, with no observable degradation, demonstrating its excellent long‐term storage stability [33].

### 2.5 Application in Human‐Machine Interaction as a Self‐Powered Sensor

**FIGURE 6:**
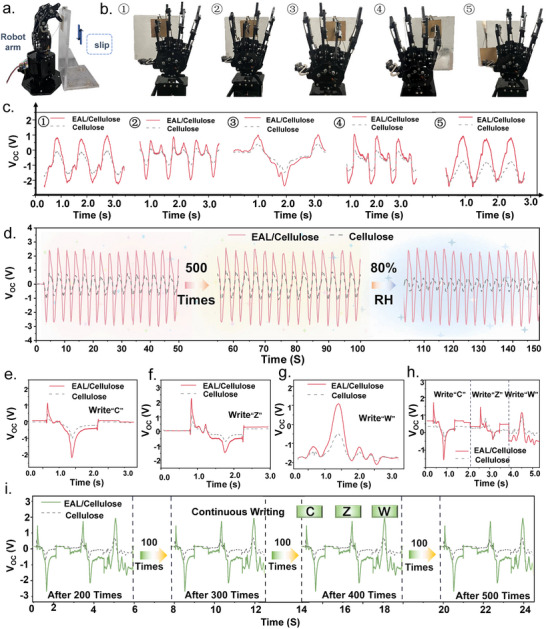
(a) Schematic illustration of a robotic arm sliding across the composite material. (b) Schematic of the robotic arm's five fingers contacting the composite surface. (c) Triboelectric‐induced electrical signals are generated by the five fingers of the robotic arm. (d) Durability evaluation through 500 continuous sliding cycles. V_OC_ corresponding to writing the letter (e) “C”, (f) “W”, and (g) “Z”. (h) V_OC_ during continuous writing of “C, Z, and W”. (i) Durability assessment under 500 continuous handwriting cycles of “CZW”.

Leveraging the high output performance and environmental stability of the EAL/Cellulose‐based TENG [34], a self‐powered sensing system was integrated into a robotic hand for behavior monitoring without external power. The TENG units were attached to the fingertip contact regions of the five robotic fingers. During sliding motions, the contact‐separation process generated triboelectric signals characteristic of each finger movement (Figure 6a, b). Due to intrinsic differences in contact area (thumb: ∼1.2 cm^2^, index: ∼0.8 cm^2^, middle: ∼0.9 cm^2^, ring: ∼0.7 cm^2^, little finger: ∼0.5 cm^2^), applied pressure, and sliding velocity, the five fingers produced distinctive electrical waveforms (Figure 6c). With a five‐channel electrode, various signals acquisition was achieved. Durability testing over 500 continuous sliding cycles demonstrated excellent stability of the EAL/Cellulose sensor, with waveform deviation below 2% and amplitude fluctuation below 5% (Figure 6d). In contrast, the pure cellulose device showed a ∼42% signal increase (from 0.72 to 1.25 V), attributed to progressive exposure of hydroxyl groups that enhanced charge transfer. Under high humidity (80% RH), the EAL/Cellulose sensor maintained stable output, while the cellulose device exhibited >60% signal decay due to water‐induced charge dissipation [35]. These results confirm that EAL modification significantly improves the robustness and humidity tolerance of triboelectric sensing interfaces [36].

To evaluate practical sensing resolution, handwriting tests were conducted using the EAL/Cellulose‐based TENG [37]. Writing the letter “C”, which involves a single continuous stroke, produced a characteristic pair of positive and negative pulses (Figure 6e). Writing “Z” resulted in a three‐stage signal consistent with its “horizontal‐diagonal‐horizontal” geometry (Figure 6f). In contrast, writing “W” generated four sets of peaks corresponding to its four alternating strokes, each showing distinct waveform profiles due to variations in writing force, displacement, and speed (Figure 6g). The unique peak numbers, pulse widths, and waveform inflection points of “C”, “Z”, and “W” enabled clear signal discrimination (Figure 6h). The pure cellulose device, with weaker triboelectric output, failed to resolve several key features, further highlighting the enhanced signal fidelity provided by the EAL/Cellulose composite. To further evaluate the durability of handwriting signal recognition, the letters “C”, “Z”, and “W” were repeatedly written 500 times. As shown in Figure 6i, the response patterns obtained after every 100 cycles closely match the corresponding shapes and characteristic features in Figure 6e–h, indicating excellent interfacial stability and reliable repeatability. These results demonstrate that the sensor can accurately track handwriting trajectories and establish a foundation for future algorithm‐based character recognition, expanding its potential in human‐machine interaction, signature verification, and soft electronics [38, 39].

### 2.6 Recyclability and Degradability of the EAL/Cellulose Composites

**FIGURE 7:**
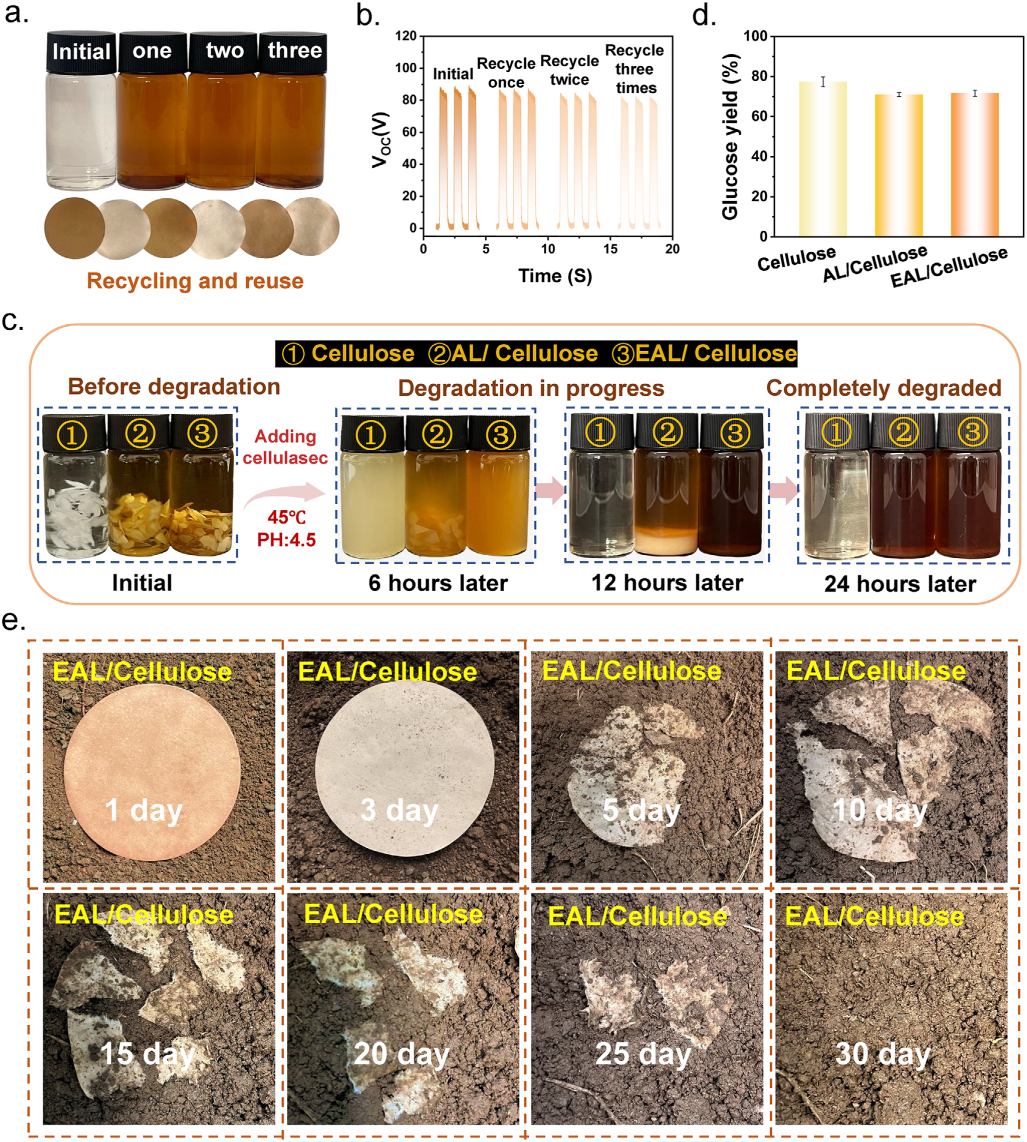
(a) Photographs of the recycling procedure for the EAL/Cellulose composite. (b) Output voltage of the EAL/Cellulose‐based TENG after three recycling cycles. (c) Enzymatic degradation process of Cellulose, AL/Cellulose, and EAL/Cellulose using cellulase. (d) Sugar content released from Cellulose, AL/Cellulose, and EAL/Cellulose after enzymatic hydrolysis. (e) Soil‐burial degradation behavior of the EAL/Cellulose composite under natural environmental conditions.

As functional composite materials intended for practical applications, recyclability and degradability are key indicators for assessing their sustainability [40, 41]. The environmental compatibility of the EAL/Cellulose composite was systematically evaluated in terms of its recycling stability and biological/natural degradability. To examine its recycling potential, a three‐cycle regeneration test was designed (Figure 7a). After the first use, the composite was dissolved in tetrahydrofuran, reassembled into a TENG device, and retested for its triboelectric output [42]. As shown in Figure 7b, the V_OC_ fluctuated by less than 5% after three recycling cycles, demonstrating excellent reprocessing stability and reliable recyclability of the EAL/Cellulose system. Enzymatic degradation tests mediated by cellulase were then employed to investigate its degradability in a controlled biological environment. The reaction conditions were set at 45°C, pH 4.5 (optimal for cellulase activity), and an enzyme concentration of 10 U/mL. As shown in Figure 7c, after 6 h of enzymatic treatment, the EAL/Cellulose suspension became uniformly turbid, indicating rapid disruption of the cellulose network. After 12 h, the composite was fully degraded, with only slight lignin floccules remaining and no visible cellulose residues. In contrast, the AL/Cellulose composite retained ∼30% undegraded cellulose under the same conditions and required up to 24 h for complete degradation, demonstrating significantly lower enzymatic efficiency. This difference can be attributed to the abundant phenolic hydroxyl groups on AL, which tend to undergo non‐specific adsorption with the active sites of cellulase, preventing the enzyme from effectively binding to the cellulose substrate and thereby suppressing catalytic activity. After esterification, the phenolic hydroxyl groups in EAL are replaced by hydrophobic ester groups, effectively avoiding such non‐productive adsorption and allowing cellulase to access the substrate efficiently, thus enhancing the degradation rate [43]. To quantitatively assess the degradation efficiency, the released reducing sugars were measured using high‐performance liquid chromatography (HPLC) (Figure 7d). After 24 h of enzymatic hydrolysis, the reducing sugar yield of the EAL/Cellulose system closely matched that of pure cellulose and was significantly higher than that of the AL/Cellulose composite, further confirming the excellent enzymatic degradability of the EAL/Cellulose material. Meanwhile, a soil‐burial test was conducted (summer, Dalian, China, 25°C–33°C, 40%–70% humidity) to evaluate the environmental degradability of the material under natural conditions. The EAL/Cellulose composite paper was buried at a depth of 10 cm, and its degradation process was monitored over 30 days. As shown in Figure 7e, the edges of the sample were almost completely degraded by day 10, and full degradation was achieved by day 30. This degradation behavior is mainly driven by dominant soil microorganisms, including actinomycetes, Bacillus species, and cellulose‐degrading fungi such as Trichoderma and Aspergillus, which secrete cellulolytic enzyme systems that depolymerize cellulose into small, assimilable sugars, thereby accelerating the breakdown of the composite structure [44]. The overall degradation rate is comparable to the natural decomposition of plant residues and effectively avoids microplastic pollution associated with conventional plastics, demonstrating the excellent environmental compatibility and sustainability of the EAL/Cellulose composite [45, 46].

## 3 Conclusion

In summary, this study successfully demonstrated that conformation engineering of lignin offers an effective molecular route for simultaneously enhancing the mechanical durability and electrical output of cellulose‐based triboelectric systems. By enabling adaptive chain mobility and efficient frictional energy dissipation, the EAL/Cellulose composite achieves a wear rate reduction of 52.44% compared with pristine cellulose‐based TENGs. Moreover, experimental measurements and theoretical calculations reveal that EAL modification increases the dielectric constant and dipole moment of lignin, thereby markedly improving the output performance from 15.48 mW m^−2^ for pristine Cellulose‐TENG to 75.89 mW m^−2^ for the optimized EAL/Cellulose TENG, with the improvement of 390%. As a potential application, the triboelectric sensor fabricated in this work exhibits reliable signal responses in robotic finger‐sliding detection and handwritten letter recognition, and maintains stable output after friction of 500 cycles, highlighting its suitability for high‐friction monitoring and smart wearable systems. The resulting material platform ensures robust sensing under repeated friction while maintaining strong recyclability and environmental compatibility. These findings underscore conformation modulation as a promising design principle for advancing durable, high‐performance, and sustainable cellulose‐based triboelectric devices.

## 4 Experimental Section

### 4.1 Chemicals and Agents

Alkali lignin (AL) was extracted from eucalyptus. Tetrahydrofuran (THF, AR grade) was purchased from Tianjin Guangfu Technology Development Co., Ltd. Oxalic acid (AR grade) was obtained from Tianjin Kemiou Chemical Reagent Co., Ltd. Succinic anhydride (SA, AR, 98%) and 1‐methylimidazole (1‐MI, 99%) were supplied by Macklin Biochemical Co., Ltd.

### 4.2 Preparation of Esterified Alkali Lignin

A mixture of alkali lignin (AL, 3 mg) and tetrahydrofuran (THF, 2 mL) was stirred at 500 rpm for 30 min to form a homogeneous dark‐brown solution. Succinic anhydride (SA, 10 g·mg^−1^ AL) and 1‐methylimidazole (1‐MI, 10 mL·mg^−1^ AL) were then added, and the reaction mixture was transferred to a round‐bottom flask and refluxed at 60°C under vigorous stirring for 3 h to complete the esterification process. After the reaction, oxalic acid was added to adjust the pH to 2, inducing precipitation of the lignin. The solid was collected by vacuum filtration and washed repeatedly with deionized water to obtain the esterified alkali lignin (EAL).

### 4.3 Preparation of EAL/Cellulose

EAL powder (0.1 g) was dispersed in 10 mL of THF and stirred for 1 h to obtain a uniform suspension. The resulting mixture was then drop‐coated onto cellulose paper (15 cm diameter) in a layer‐by‐layer manner, followed by drying at room temperature after each coating step to form the EAL/Cellulose composite.

### 4.4 Assembly of EAL/Cellulose‐Based TENG Device

An EAL/Cellulose film and a PVDF film (5 × 5 cm^2^) were each attached to silver conductive tape and used as the tribo‐positive and tribo‐negative layers, respectively. The two layers were then aligned in a vertical contact–separation configuration, and the output performance of the assembled TENG was characterized under this operating mode.

### 4.5 Characterizations

The surface potential of the films was characterized using atomic force microscopy (AFM) equipped with a Kelvin probe force microscopy (KPFM) mode. The microstructure and elemental distribution were examined using a scanning electron microscope (SEM, Hitachi S‐4800) coupled with an energy‐dispersive spectroscopy (EDS) system. Fourier transform infrared (FTIR) spectra were collected using a Varian 800 FTIR spectrometer. X‐ray photoelectron spectroscopy (XPS) measurements were performed on a Thermo Fisher instrument equipped with a Kα X‐ray source to analyze the binding energy spectra. Mechanical properties were evaluated at room temperature using a universal testing machine (Instron 5965, Instron, USA) at a tensile rate of 25 mm min^−1^. The dielectric constant was measured at room temperature using an Agilent LCR meter (Model 4990A) with frequencies ranging from 1 kHz to 1 MHz. The electrical output performance of the TENG devices was tested by applying periodic external forces using a commercial linear motor (LinMot). The open‐circuit voltage (V_OC_), short‐circuit current (I_SC_), and transferred charge (Q_SC_) were recorded using a Keithley 6514 electrometer equipped with a data acquisition card. The output power of the TENG was evaluated by connecting external load resistances ranging from 1 kΩ to 400 MΩ. The triboelectric potential distribution was simulated using COMSOL Multiphysics software. The friction coefficient was measured using an Anton Paar TRB3 tribometer (Switzerland) under conditions of 3 N normal load, 3 Hz sliding frequency, and a stroke length of 2 mm. Surface depth profiles were obtained using an Alpha‐Step IQ surface profilometer, and wear volume was estimated using the classical wear equation. For self‐powered sensing measurements, handwritten letter signals were collected using a Keithley 6514 electrometer and an NI data acquisition card (National Instruments, USA) with a load resistance of 100 MΩ.

### 4.6 Statistical Analysis

All experimental data are presented as mean ± standard deviation (SD), and each measurement was repeated at least three times unless otherwise specified. Statistical analysis, curve fitting, and data visualization were performed using Origin 2021. Error bars in the figures represent the standard deviation of independent measurements.

## Conflicts of Interest

The authors declare no conflict of interest.

## Supporting information




**Supporting File**: advs73715‐sup‐0001‐SuppMat.docx.

## Data Availability

The data that support the findings of this study are available from the corresponding author upon reasonable request.
