# Supplementary material for: Conformation‐Modulated Lignin for Durable and High‐Output Cellulosic Triboelectric Materials Toward Self‐Powered Sensing
Source: Adv Sci (Weinh). 2026 Feb 9;13(16):e73715. doi: 10.1002/advs.73715 (PMC13042924; doi:10.1002/advs.73715)
Supplement: Supplementary file 1 — Supporting File: advs73715‐sup‐0001‐SuppMat.docx. [file ADVS-13-e73715-s001.docx]

Supporting Information

Conformation-Modulated Lignin for ****Mechanically**** Durable and High-Output Cellulosic Triboelectric Materials toward Self-Powered Sensing

Lujie Wang^a^, Jian Du^a,c,^*, Yilin Wang^a^, Tianshuang Bao^a^, Chao Li^a,c^, Yehan Tao^a^, Jinwen Hu^a^, Chenglong Fu^a^, Dong Lv^d^, Weiwei Zhao^e,^*, Zhanhui Yuan^b,^*, Haisong Wang^a,^*

^a^Liaoning Key Lab of Lignocellulose Chemistry and BioMaterials, Liaoning Collaborative Innovation Center for Lignocellulosic Biorefinery, College of Light Industry and Chemical Engineering, Dalian Polytechnic University, Dalian, 116034, China

^b^College of Materials Engineering, Fujian Agriculture and Forestry University, Fuzhou 350108, China

^c^Department of Materials Science and EngineeringPohang University of Science and Technology77 Cheongam-Ro, Nam-Gu, Pohang 37673, Republic of Korea

^d^School of Energy and Environment, City University of Hong Kong, Hong Kong, 999077, China

^e^Birmingham Centre for Energy Storage (BCES) & School of Chemical Engineering, University of Birmingham, Birmingham, B15 2TT, UK

*Corresponding authors: dujian01@dlpu.edu.cn (Jian Du); w.zhao@bham.ac.uk (Weiwei Zhao); zhanhuiyuan@fafu.edu.cn (Zhanhui Yuan); wanghs@dlpu.edu.cn (Haisong Wang)


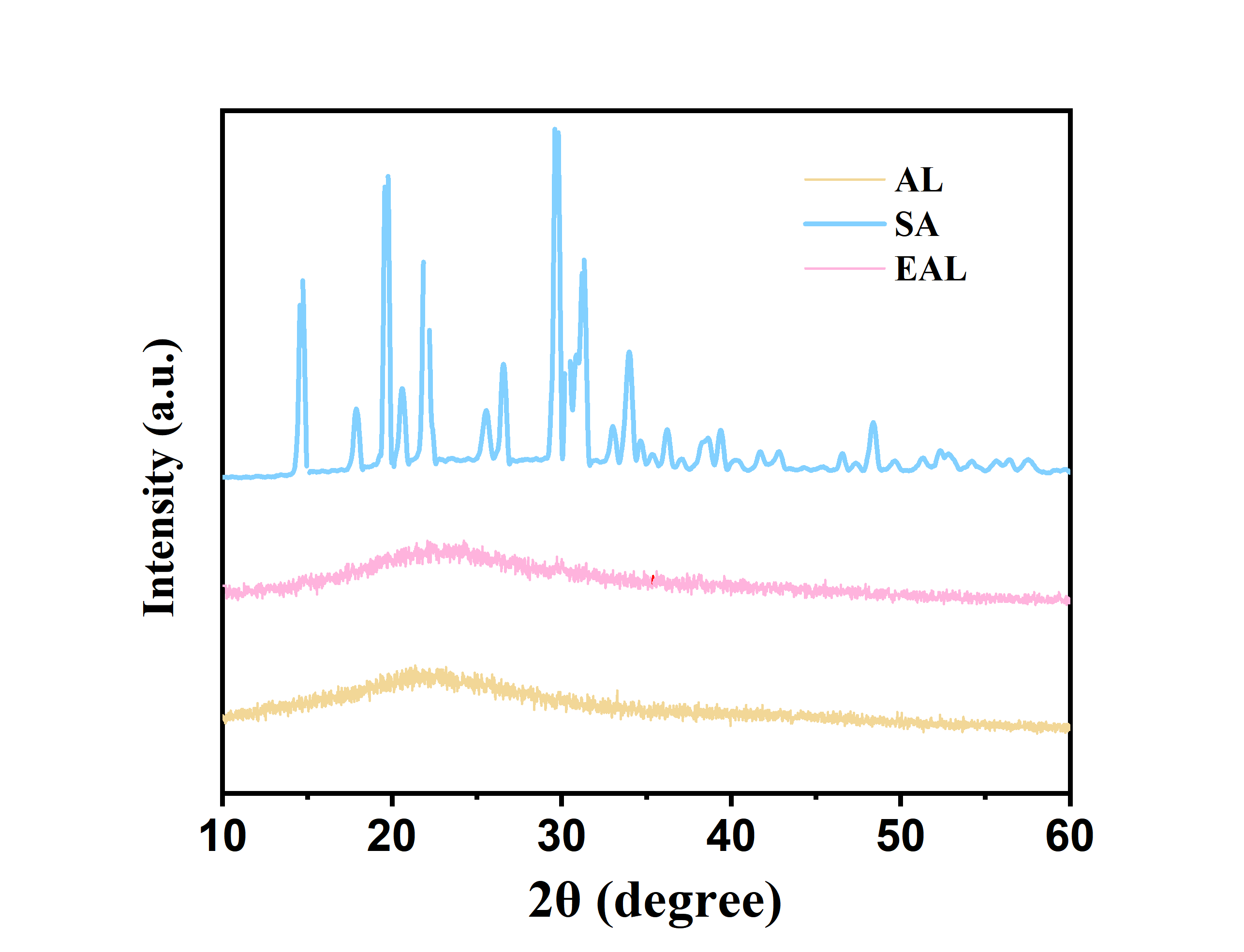


**Figure. S1** XRD patterns of AL, SA and EAL.


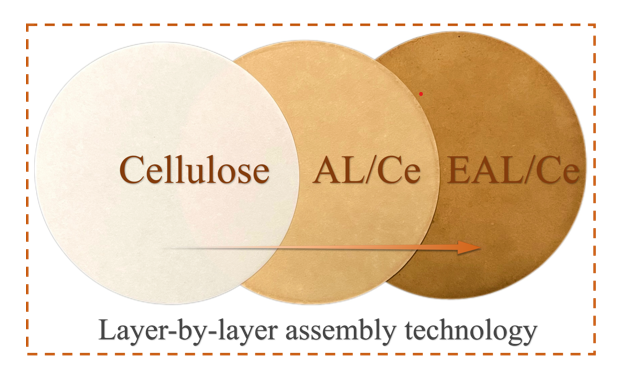


**Figure. S2** Photographs of Cellulose, AL/Cellulose, EAL/Cellulose.

(a)


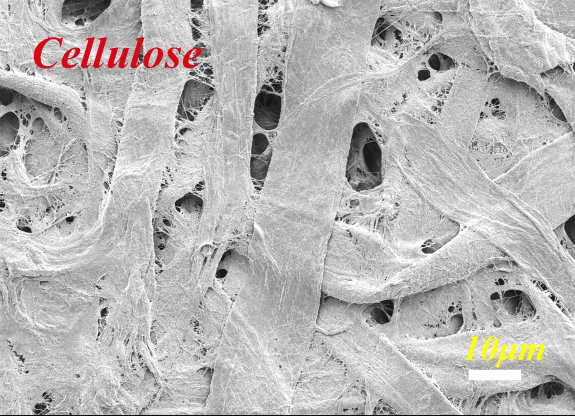


(b)


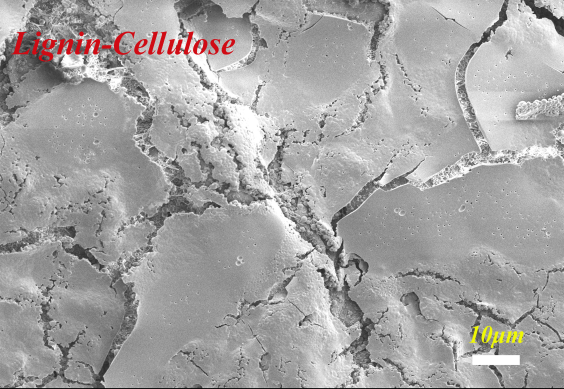


(c)


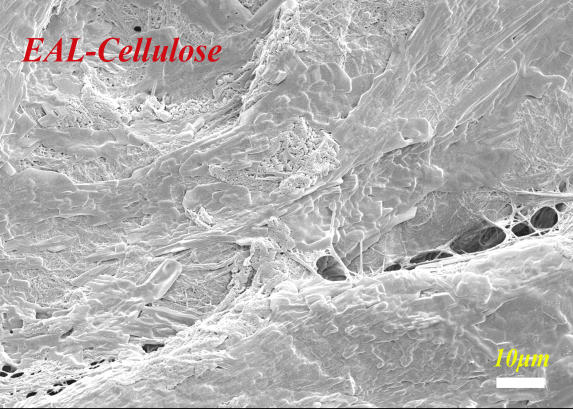


**Figure. S3** SEM images of (a) Cellulose and (b) AL/Cellulose and (c) EAL/Cellulose.


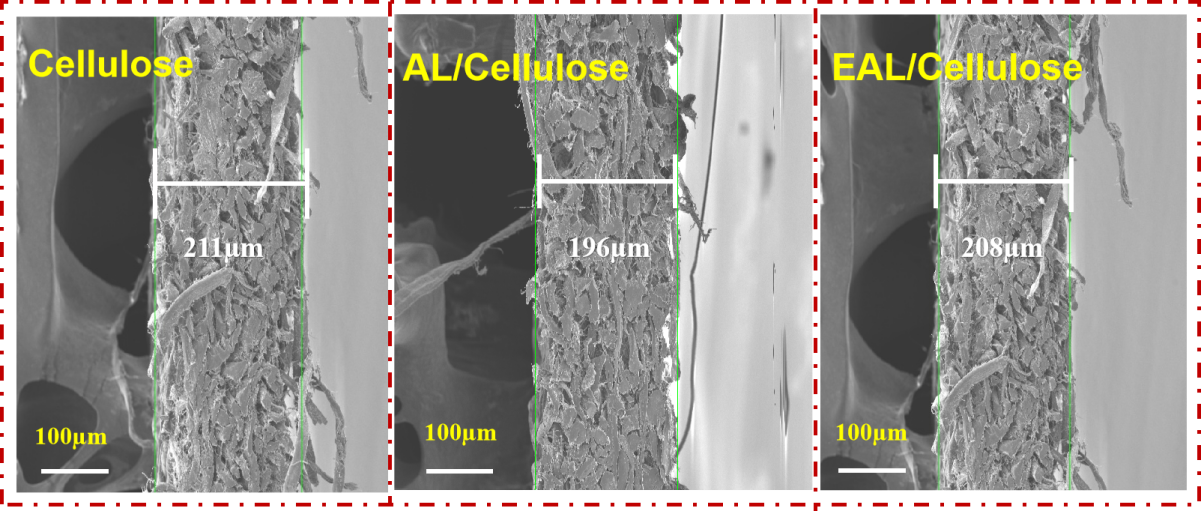


**Figure. S4** Cross-sectional SEM images of cross-sections of Cellulose, AL/Cellulose, and EAL/Cellulose.

(b)

(a)

(c)


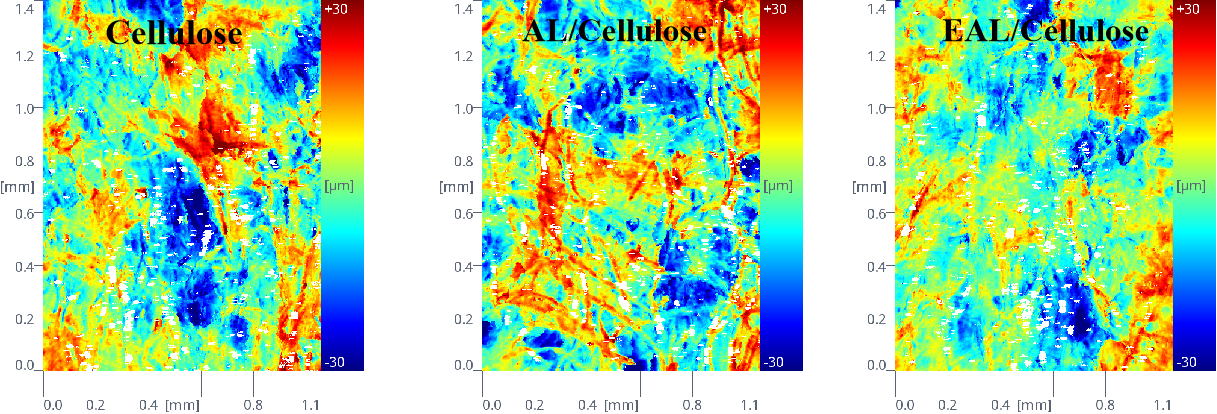


**Figure. S5** 2D dimensional surface morphology images of (a) Cellulose, (b) AL/Cellulose, (c) EAL/Cellulose.


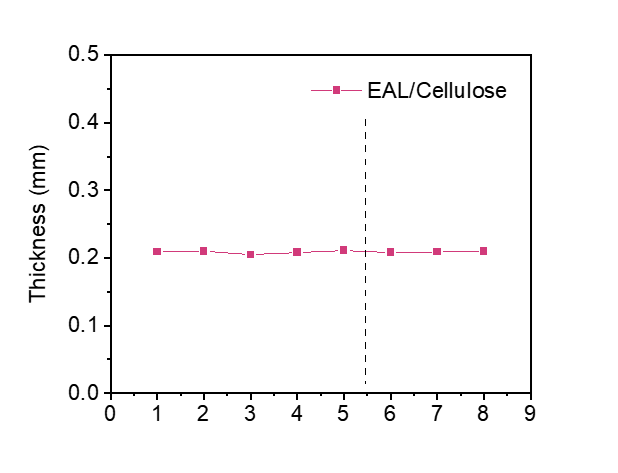


**Figure. S6** EAL/Cellulose paper thickness measurement diagram. The obtained thickness values were 0.209, 0.210, 0.205, 0.208, and 0.211 mm (points of 1-5), respectively, yielding an average of 0.209 ± 0.002 mm (mean ± SD). The average thicknesses of the three batches were 0.209 mm, 0.208 mm, and 0.210 mm (points of 6-8), respectively, with an interbatch standard deviation of only 0.001 mm.


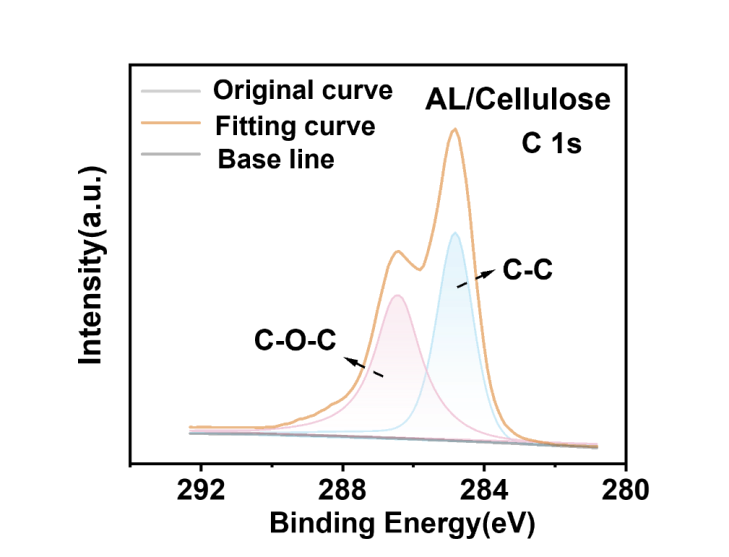


**Figure. S7** XPS C1s image AL/Cellulose.


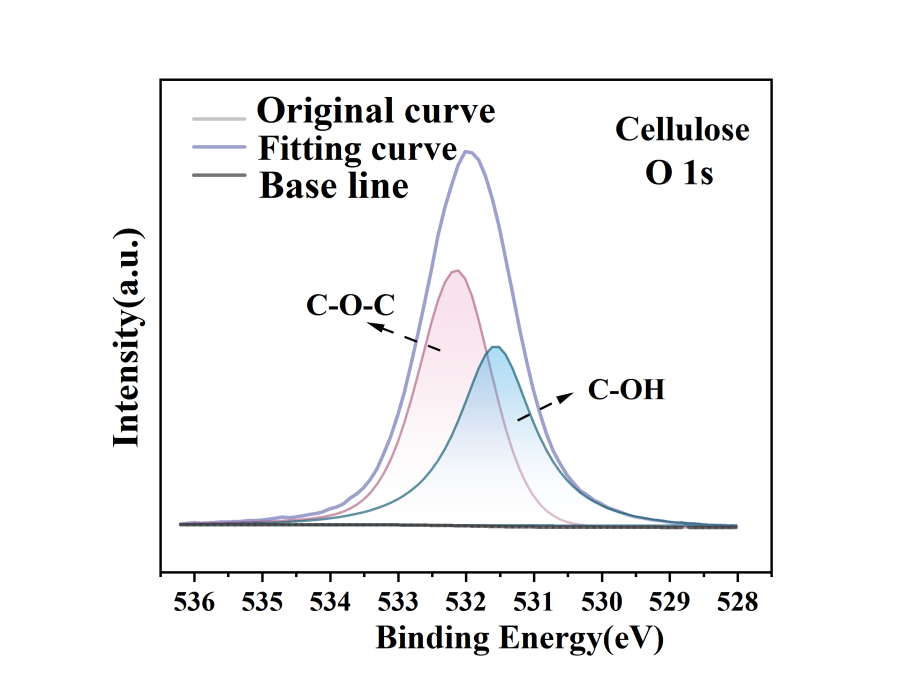

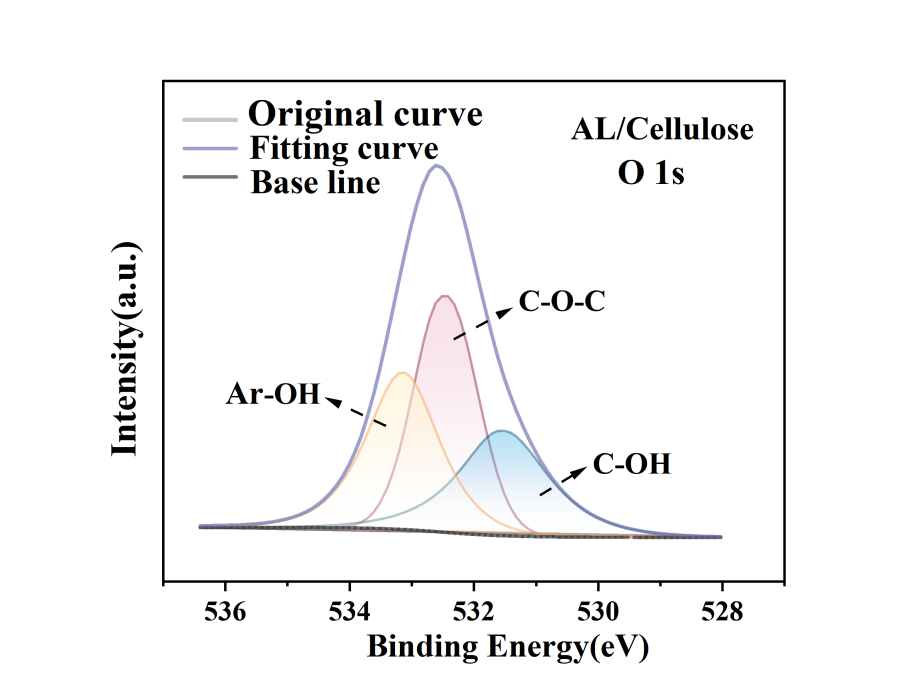

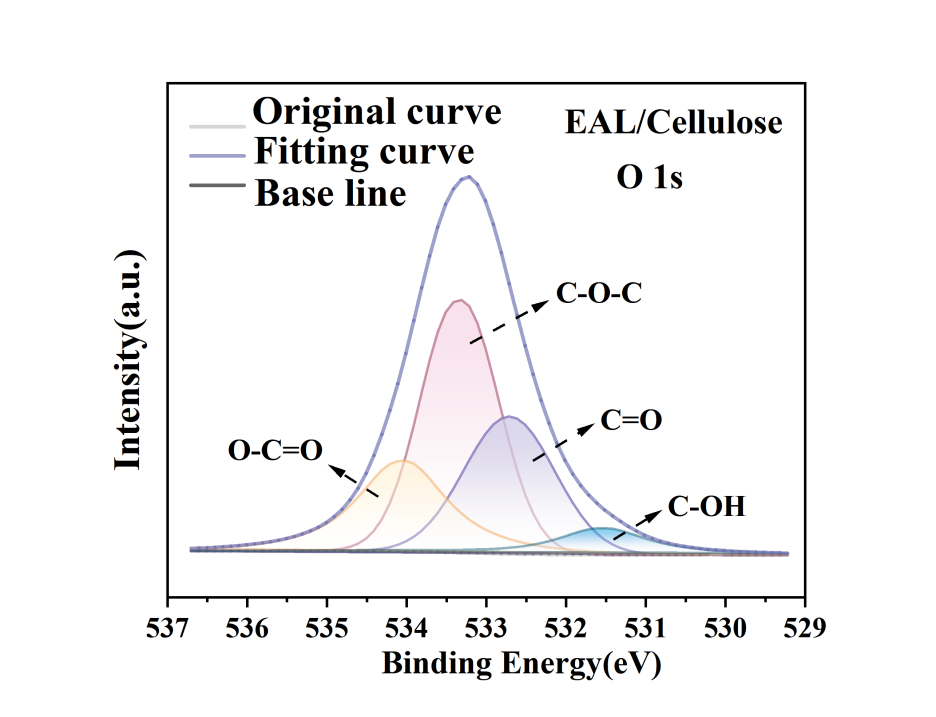


(c)

(b)

(a)

**Figure. S8** XPS O1s image of (a) Cellulose and (b) AL/Cellulose and (c) EAL/Cellulose.


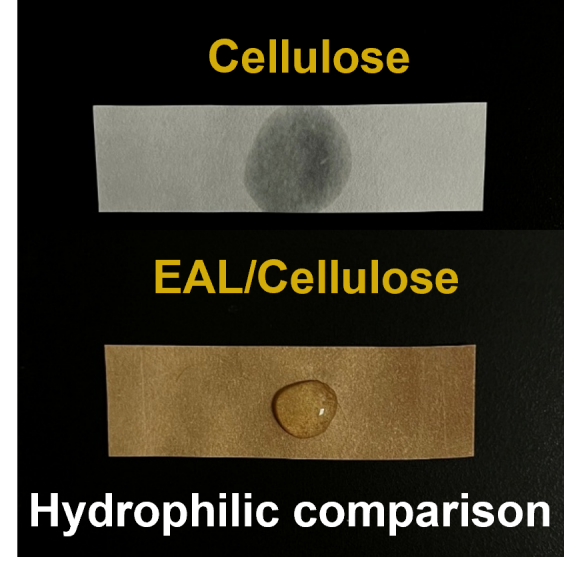


**Figure. S9** Photographs of water infiltration on Cellulose and EAL/Cellulose.


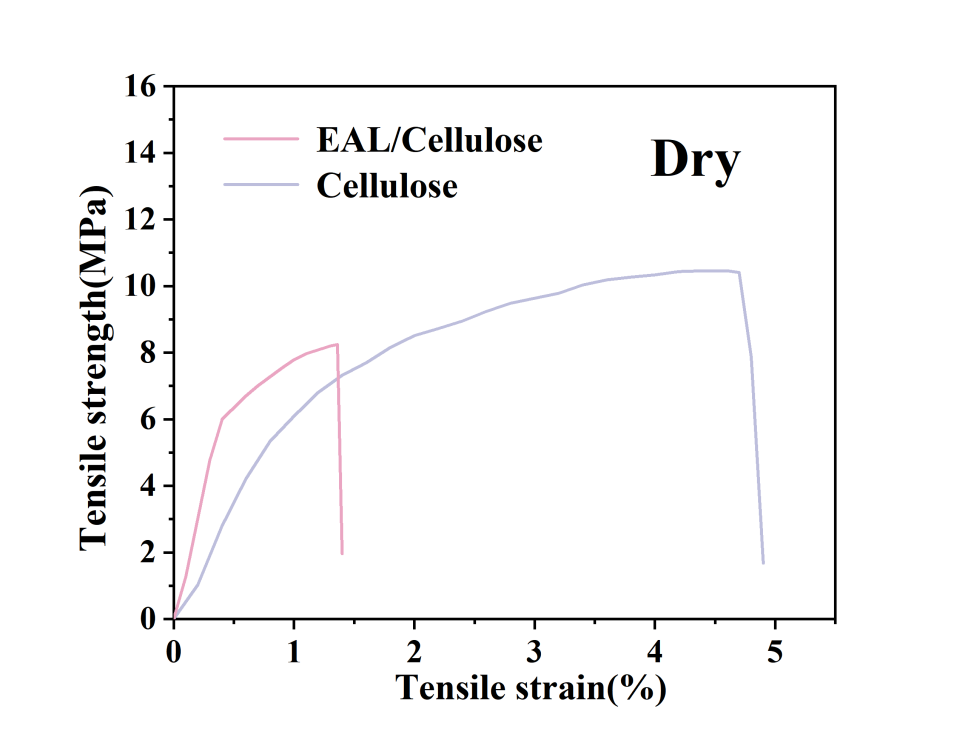

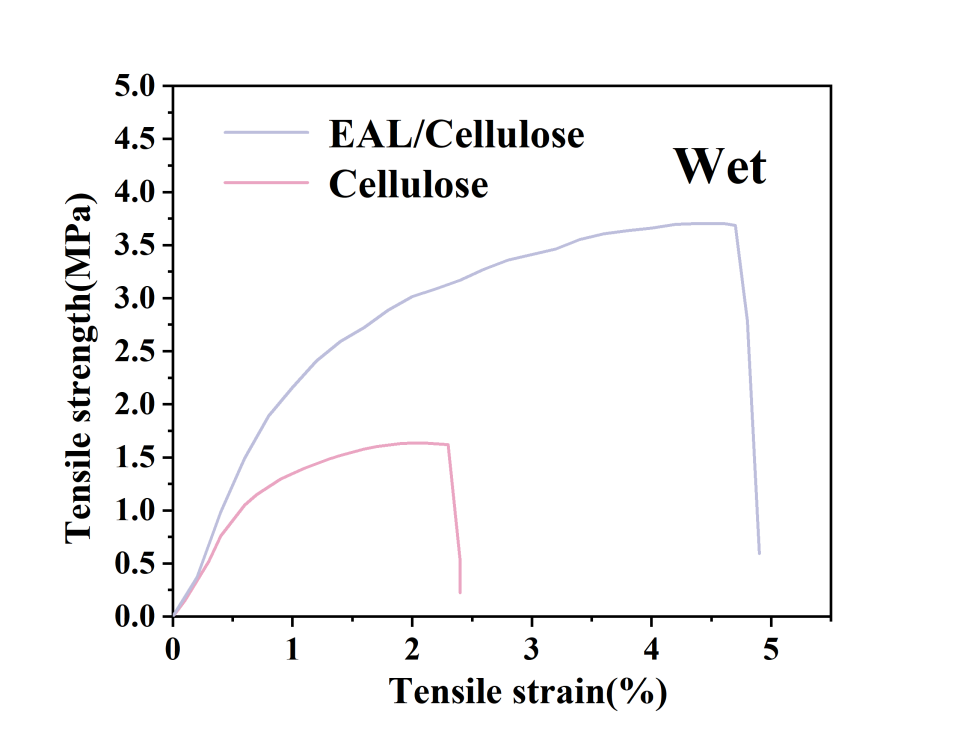


(b)

(a)

**Figure. S10** (a) Dry and (b) wet tensile properties of cellulose and EAL/Cellulose.


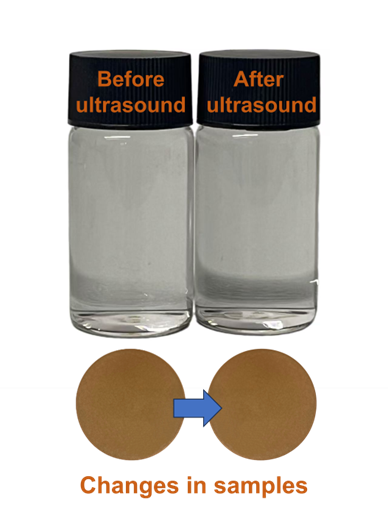


**Figure. S11** Comparison of EAL/Cellulose paper before and after ultrasound treatment (300 W, 40 kHz) for 60 minutes.


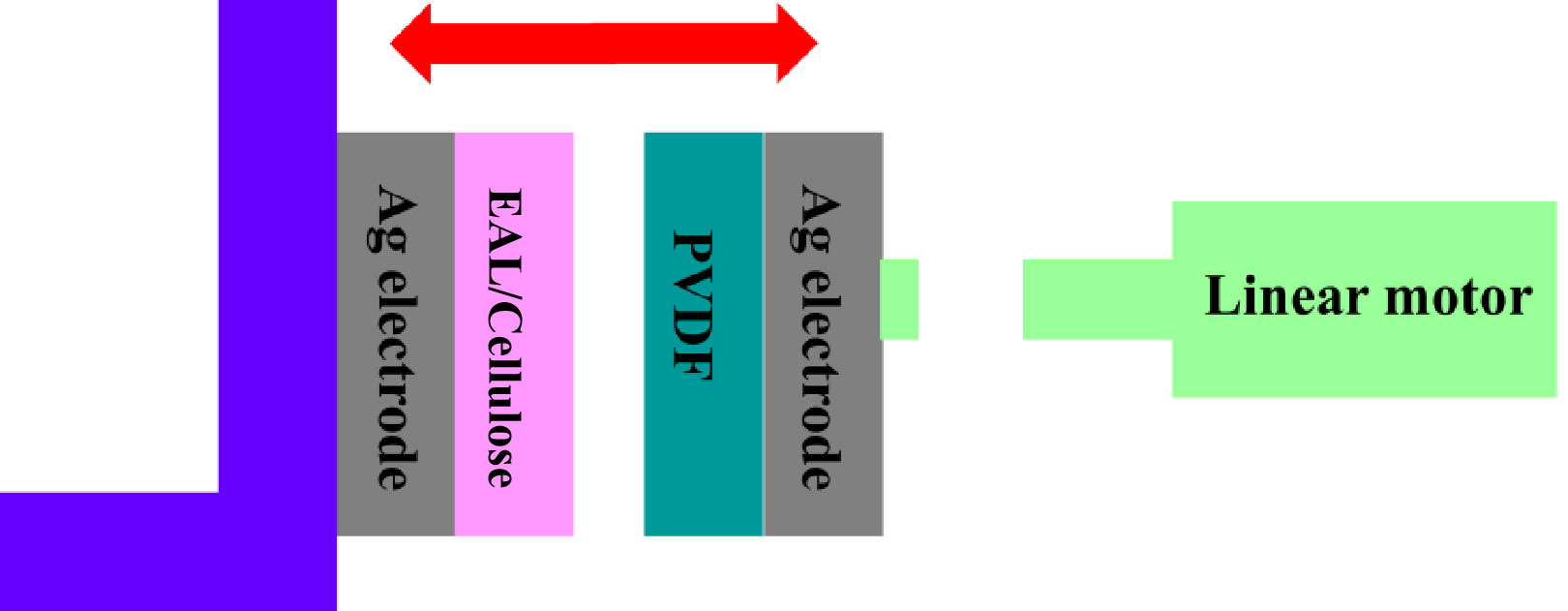


**Figure. S12** Schematic diagram of electrode movement measurement.


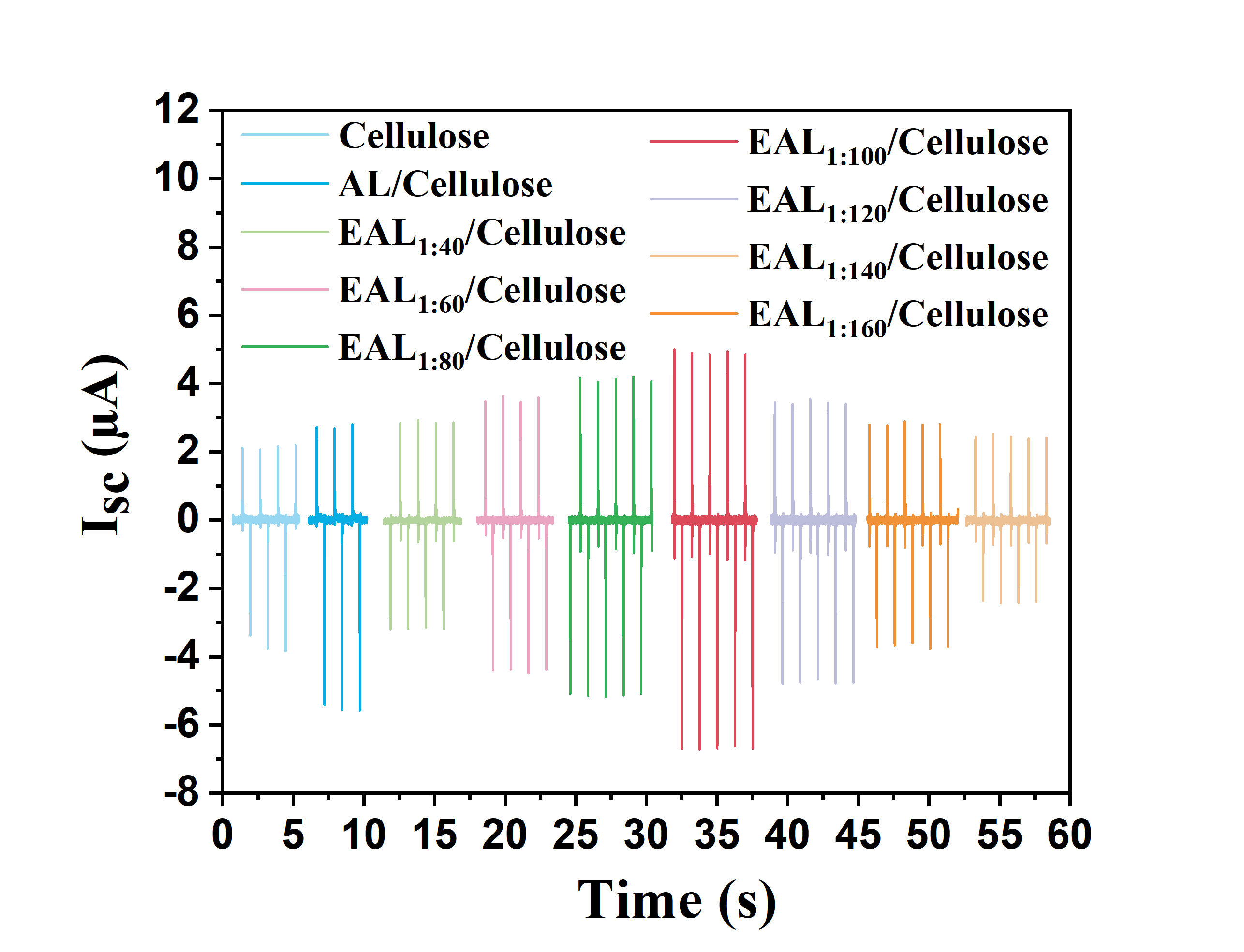

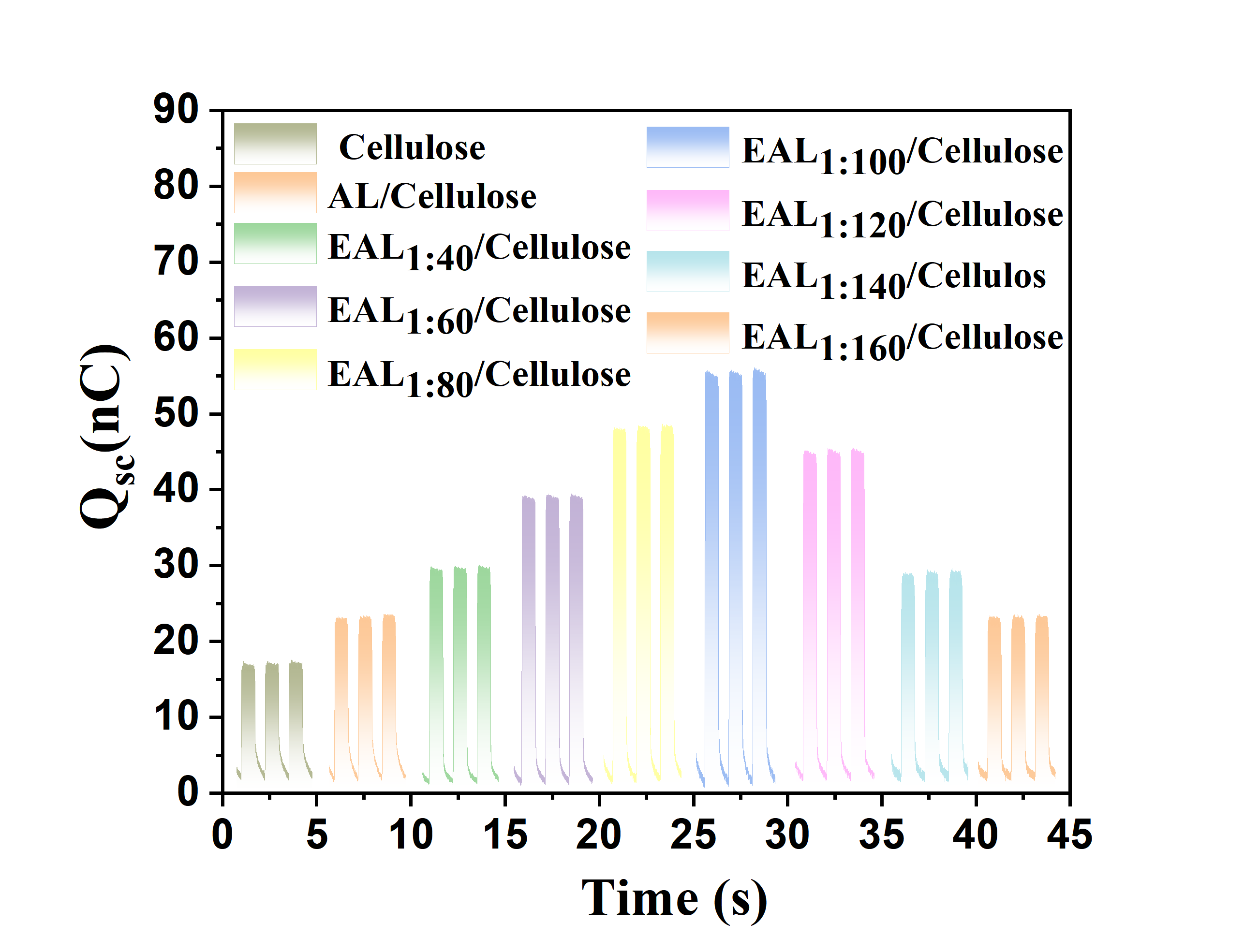


(b)

(a)

**Figure. S13** (a) I_SC_ and (b) Q_SC_ of EAL/Cellulose as the function of SA doping amount.


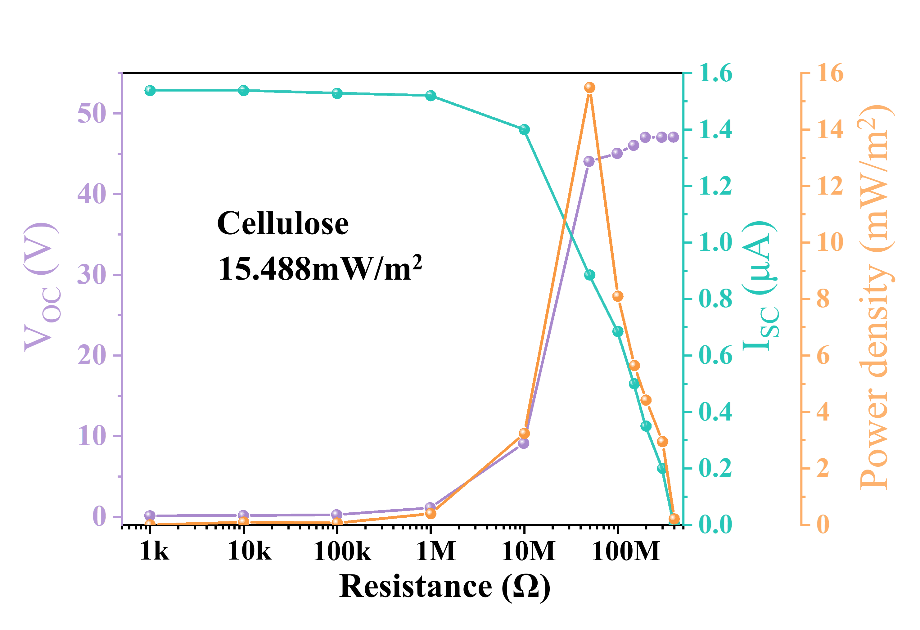

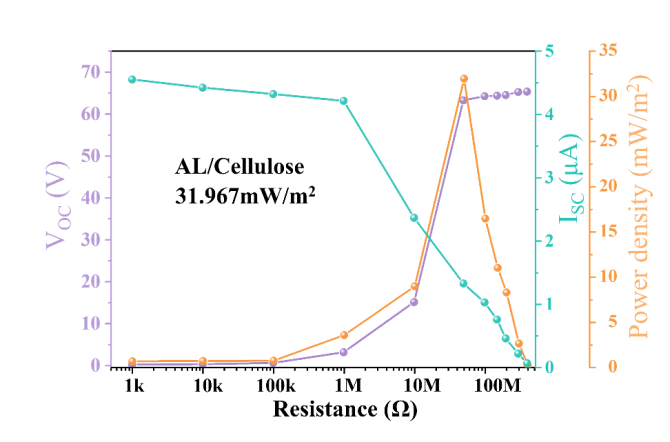


(b)

(a)


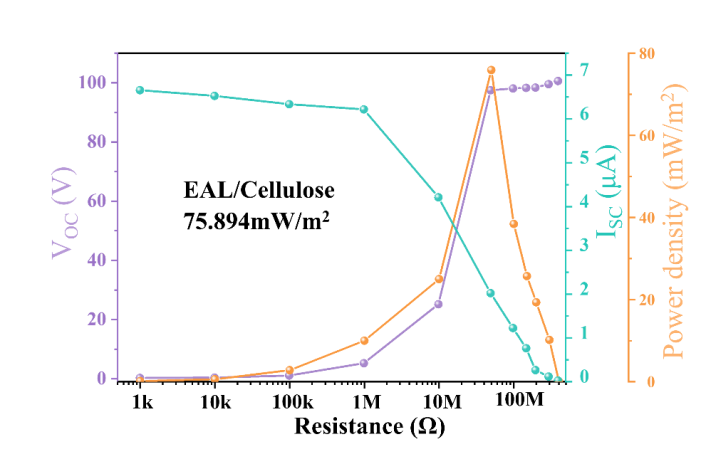


(c)

**Figure. S14** Output power density maps of (a) Cellulose, (b) AL/Cellulose, (c) EAL/Cellulose.


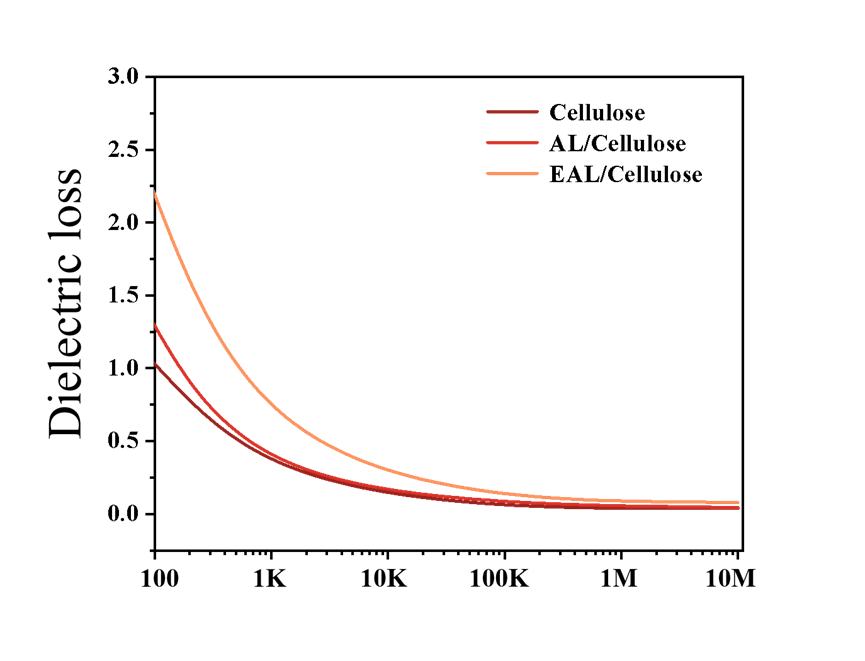


**Figure. S15** Dielectric loss of Cellulose, AL/Cellulose, EAL/Cellulose.


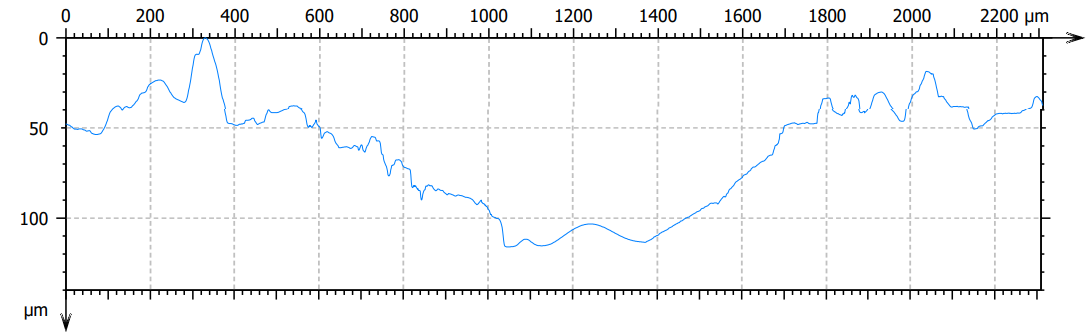


**Figure. S16** Wear depth after friction of pristine Cellulose.


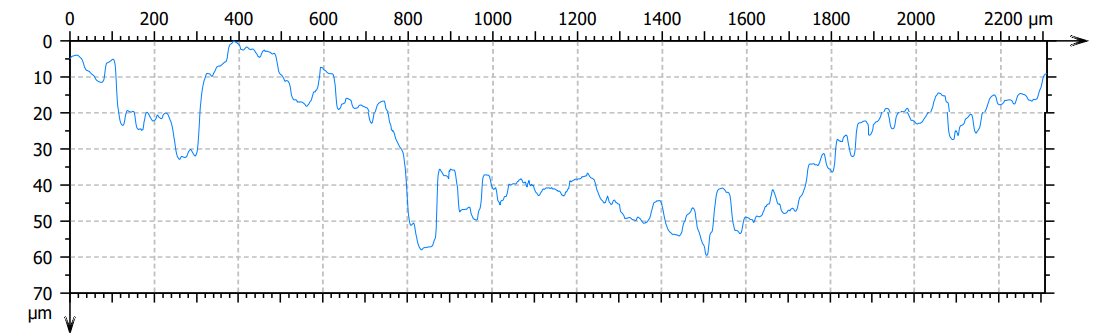


**Figure. S17** Wear depth after friction of AL/Cellulose.


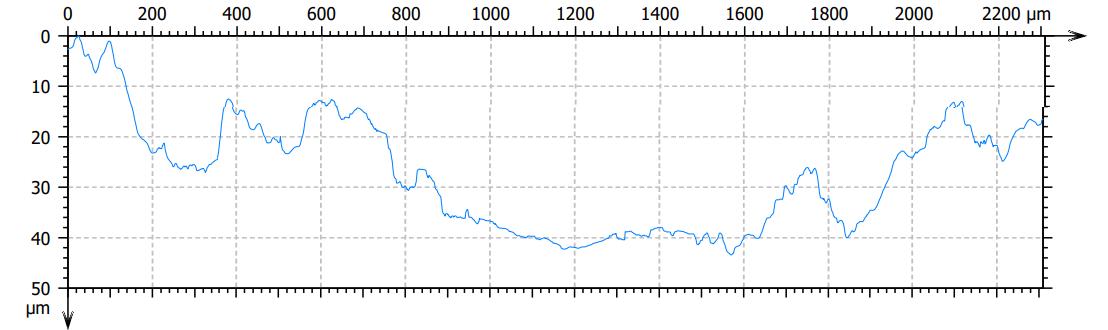


**Figure. S18** Wear depth after friction of EAL/Cellulose.

**Table S1** Output performance comparison of cellulose paper-based TENG.

| Triboelectric positive material | Triboelectric negative material | Electrode size (cm^2^) | V_OC_（V） | I_SC_ (μA) | Reference |
| --- | --- | --- | --- | --- | --- |
| EAL/Cellulose paper | PVDF | 25 | 100 | 5.26 | This work |
| Cellulose/BaTiO_3_ paper | PDMS | 4 | 48 | 5.1 | [1] |
| Superhydrophobic cellulose paper | PTFE | 16 | 21.6 | 2.8 | [2] |
| CNF paper | AgNW | 25 | 21 | 2.5 | [3] |
| Lignin/polycaprolactone nanofiber | VHB | 16 | 95 | - | [4] |
| Cellulose/BT paper | PDMS | 25 | 88 | 8.3 | [5] |
| PEI-paper | PTFE | 8 | 68 | 4.47 | [6] |
| Cellulose film | PET | 1 | 97 | 5.7 | [7] |
| Enzymatic paper | PVDF | 25 | 90.5 | 4.7 | [8] |

**Table S2** Output performance comparison of previously reported wear-resistant triboelectric materials.

| Wear-resistant material | Electrode size (cm^2^) | V_OC_ (V） | I_SC_ (μA) | Resistance | Reference |
| --- | --- | --- | --- | --- | --- |
| B_4_C/PVDF | 16 | 155.4 | 7.9 | YES | [9] |
| ZnO/CNTs/GO/PPS/LCP | 8 | 27.9 | 0.93 | YES | [10] |
| SCF-COOH-UIO66/PPS | 4 | 7.2 | 1.8 | YES | [11] |
| OA-PS | 8 | 355 | 9.4 | YES | [12] |
| DCL-glass | 6.25 | 400 | - | YES | [13] |
| SA-PTFE/PVDF-HFP | 4 | 145 | 18 | YES | [14] |
| IGNs/Fe_3_O_4_/PI | - | 65 | 2.48 | YES | [15] |
| EAL/Cellulose paper | 25 | 100 | 5.86 | YES | This work |

**References**

1. K. Shi, X. Huang, B. Sun, Z. Wu, J. He, P. Jiang, Cellulose/BaTiO3 aerogel paper based flexible piezoelectric nanogenerators and the electric coupling with triboelectricity, *Nano Energy*, 57 (2019) 450-458.
2. S Nie, H Guo, Y Lu, et al. Superhydrophobic cellulose paper‐based triboelectric nanogenerator for water drop energy harvesting. *AMT*. (2020), 5(9): 2000454.
3. C Yao, A Hernandez, Y Yu, et al. Triboelectric nanogenerators and power-boards from cellulose nanofibrils and recycled materials. *Nano Energy*, (2016), 30: 103-108.
4. H. Jo, D. Park, M. Joo, D. Choi, J. Kang, J. Ha, K. Kim, K. Kim, S. An: Performance-enhanced eco-friendly triboelectric nanogenerator via wettability manipulation of lignin. *ECOMAT.* (2023) 5.
5. K. Shi, H. Zou, B. Sun, P. Jiang, J. He, X. Huang, Dielectric modulated cellulose paper/PDMS-based triboelectric nanogenerators for wireless transmission and electropolymerization applications, *Adv. Funct. Mater*. 30 (2019) 1904536.
6. S. Wu, G. Li, W. Liu, D. Yu, G. Li, X. Liu, Z. Song, H. Wang, H. Liu, Fabrication of polyethyleneimine-paper composites with improved tribopositivity for triboelectric nanogenerators, *Nano Energy.* 93 (2022) 106859.
7. J. Du, C. Jiao, C. Li, Y. Tao, J. Lu, Y. Cheng, X. Xia, M. Tan, H. Wang, Eco-friendly and humidity-sensitive cellulosic triboelectric materials tailored by xylanase for monitoring the freshness of fruits, *Nano Energy.* 116 (2023) 108803.
8. Y. Chen, D. Li, Y. Xu, Z. Ling, H. Nawaz, S. Chen, F. Xu: Surface-microstructured cellulose films toward sensitive pressure sensors and efficient triboelectric nanogenerators. *International Journal of Biological Macromolecules.* 208 (2022) 324-332.

[9] Y. Wang, X. Cao, N. Wang, B_4_C/PVDF-based triboelectric nanogenerator: Achieving highwear-resistance and thermal conductivity: *Tribology International.* (2024) 197 109828.

[10] K. Xu, T. Peng, B. Zhang, Y. Wu. Z. Huang, Q. Guan, Zinc oxide bridges the nanofillers to enhance the wear resistance andstability of triboelectric nanogenerators. *Chemical Engineering Journal.* (2024) 493 152532.

[11] K. Xu, B. Zhang, S. Guan, Z. Huang, X. Pei, Q. Guan, Wear-resistance triboelectric nanogenerator based on metal-organicframework modified short carbon fiber reinforced polyphenylene sulfide. *Chemical Engineering Journal.* (2024) 501 157781.

[12] J. Zhang, Y. Zheng, L. Xu, D. Wang, Oleic-acid enhanced triboelectric nanogenerator with high output performance and wear resistance. *Nano Energy.* (2020) 69 104435.

[13] W. Li, L. Lu, C. Zhang, K. Loos, Y. Pei, Durable and high-performance triboelectric nanogenerator based on an inorganic triboelectric pair of diamond-like-carbon and glass. *Advanced Science.* (2024) 11 2309170.

[14] M. Qu, H. Liu, Y. Xue, J. Li, Q. Liu, J. Yan, Y. Zhao, L. Mu, C. Sun, J. He. Stearic acid-enhanced triboelectric nanogenerators with high waterproof, output performance, and wear resistance for efficient harvesting of mechanical energy and self-powered sensing for human motion monitoring. *ACS Applied Electronic Materials.* (2024) 6 1651-1665.

[15] Z. Cao, X. Xie, X. Chen, J. Yu, X. Liu, Y. Huang, X. Xu, S. Lu, Y. Li. Wear- and high-temperature-resistant IGNs/ Fe_3_O_4_/PI composites for triboelectric nanogenerator. *Journal of Electronic Materials.* (2022) 51 4986-4994.
